# UGT1A1 and Sacituzumab Govitecan Toxicity: A Systematic Review and Meta‐Analysis

**DOI:** 10.1002/cpt.70060

**Published:** 2025-09-11

**Authors:** Cinzia Dello Russo, Innocent Gerald Asiimwe, Sudeep Pushpakom, Carlo Palmieri, Munir Pirmohamed

**Affiliations:** ^1^ Department of Pharmacology and Therapeutics, Institute of Systems, Molecular and Integrative Biology University of Liverpool Liverpool UK; ^2^ Section of Pharmacology, Department of Healthcare Surveillance and Bioethics Università Cattolica del Sacro Cuore ‐ Fondazione Policlinico Universitario A. Gemelli, IRCCS Rome Italy; ^3^ Department of Health Data Science, Institute of Population Health University of Liverpool Liverpool UK; ^4^ The Wolfson Centre for Personalised Medicine, Centre for Drug Safety Science University of Liverpool Liverpool UK; ^5^ Department of Molecular and Clinical Cancer Medicine, Institute of Systems, Molecular and Integrative Biology University of Liverpool Liverpool UK; ^6^ The Clatterbridge Cancer Centre NHS Foundation Trust Liverpool UK

## Abstract

Sacituzumab govitecan (SG), a humanized antibody‐drug conjugate, enables intra‐tumor delivery of SN‐38, the active metabolite of irinotecan, with the aim of increasing efficacy. SN‐38 is predominantly inactivated by the polymorphically expressed uridine diphosphate glucuronosyltransferase 1A1 (UGT1AA) where reduced activity can lead to toxicity. SG toxicity closely resembles that of irinotecan. We conducted a systematic review and meta‐analysis (PROSPERO ID: CRD42024598820) to assess *UGT1A1* genotype as a determinant of SG toxicity. Studies published up to September 29, 2024, on *UGT1A1* genotype and SG toxicity were eligible. Risk of bias was assessed using the STROPS guideline. Effect estimates for each genotype, comparing heterozygotes and homozygotes to wild‐type, were analyzed. Odds ratios (ORs) with 95% Confidence Intervals (CIs) and forest plots were generated for each exposure‐outcome combination. Four clinical trials including 999 *UGT1A1* genotyped subjects were selected for the meta‐analysis. SG treatment in UGT1A1*28 homozygous subjects increased the risk of toxicity. The OR (95% CI) was 1.80 (1.03–3.14) for neutropenia, 1.38 (0.90–2.10) for diarrhea, and 1.62 (1.07–2.45) for anemia of any grade, with low heterogeneity (*I* ^*2*^ ≤ 28%). The OR for all severe (grade ≥ 3) toxicities combined was 7.03 (95% CI: 3.41–14.50, *I* ^*2*^ = 18%). UGT1A*28 homozygous subjects were more likely to have dose reductions and treatment interruptions compared to wild‐type individuals. In conclusion, individuals with UGT1A*28/*28 genotype are at an increased risk of severe SG‐related toxicity. Pre‐treatment genotyping should be used to identify individuals that may benefit from personalized dosing, closer monitoring or alternative therapies.

**Study Highlights**

**WHAT IS THE CURRENT KNOWLEDGE ON THE TOPIC?**

Sacituzumab Govitecan (SG), a humanized antibody‐drug conjugate targeting the surface glycoprotein Trop‐2, has been engineered to enhance the intra‐tumor delivery of SN‐38 and reduce its systemic toxicity. However, toxicity closely resembling those of irinotecan emerged during drug development suggesting the involvement of genetic variation in *UGT1A1*. Current advice states that individuals with the UGT1A1*28/*28 genotype should be monitored closely, but they do not recommend pre‐prescription genotyping and there are no recommendations on dose modification in individuals who have reduced UGT1A1 activity.

**WHAT QUESTION DID THIS STUDY ADDRESS?**

We conducted a systematic review and meta‐analysis to understand whether *UGT1A1* genotype acts as determinant of SG toxicity.

**WHAT DOES THIS STUDY ADD TO OUR KNOWLEDGE?**

Our analysis combining data from four trials shows a significantly increased risk of any grade and severe forms of toxicity in UGT1A1*28 homozygous subjects treated with SG. Importantly, the OR for all severe (grade ≥ 3) toxicities combined was 7.03 (95% CI 3.41–14.50, *I* ^2^ = 18%). UGT1A*28 homozygous subjects were more likely to have dose reductions and treatment interruptions compared to wild‐type individuals.

**HOW MIGHT THIS CHANGE DRUG DISCOVERY, DEVELOPMENT, AND/OR THERAPEUTICS?**

Currently, the SG drug label advises a 25% dose reduction with the first occurrence of severe toxicities, and to withhold or permanently discontinue the drug in patients with severe adverse reactions, but does not advocate pre‐prescription *UGT1A1* genotyping. Our data suggests that pre‐treatment *UGT1A1* genotyping should be implemented in clinical practice, identifying individuals who would benefit from a personalized therapeutic approach, for example, an initial 25% dose reduction.

Sacituzumab govitecan (SG, IMMU‐132) is a humanized (IgG1k) antibody‐drug conjugate (ADC) that targets the surface glycoprotein Trop‐2 (trophoblast cell‐surface antigen) allowing for the intra‐tumor delivery of SN‐38, the active metabolite of irinotecan.^1^ The latter is a semi‐synthetic derivative of camptothecin, which is extracted from the bark of *Camptotheca acuminata*.^2^

Irinotecan is a topoisomerase I inhibitor.^2^ It is rapidly metabolized in the liver and intestine by carboxylesterases to SN‐38, a metabolite that is 100 to 1,000‐fold more active than irinotecan.^3^ SN‐38 is further detoxified by the uridine diphosphate glucuronosyltransferase (UGT) enzyme, mainly the UGT1A1 isoform.^3^ Several *UGT1A1* allelic variants have been characterized,^4^ either in the promoter or the coding region (Exon 5) of the gene, that can reduce protein expression and activity.^5^ The variant allele frequency varies across different populations: UGT1A1*28 is most frequently found in the Caucasian and African populations, while the *6 variant is found in Asians.^5^

UGT1A1 poor metabolizers are at increased risk of severe toxicity, particularly neutropenia and diarrhea, when treated with a standard dose of irinotecan.^3^ Currently available pharmacogenetic prescribing guidelines, including those issued by the Dutch Pharmacogenetics Working Group,^3^ the Italian Society of Medical Oncology and the Italian Society of Pharmacology^6^ and the French National Network of Pharmacogenetics,^5^ recommend a 30% reduction in the initial dose of irinotecan in UGT1A1 poor metabolizers to reduce toxicity. Similar recommendations have been made by different regulatory agencies, although the drug labels of irinotecan medicinal products do not mandate pre‐treatment *UGT1A1* genotyping.

SG has been engineered to enhance the intra‐tumor delivery of SN‐38 and reduce its systemic toxicity. Trop‐2, the target of this antibody, is expressed in a variety of epithelial tumors that are associated with aggressive phenotypes.^7^ The CL2A linker which is attached to the hydroxyl group on the SN‐38’s lactone ring presents a pH‐dependent cleavage site, leading to the release of SN‐38 at low pH in lysosomes and in the tumor microenvironment.^7^

In the first‐in‐human clinical study, IMMU‐132‐01 (NCT01631552), a single arm, multicenter phase 1/2 basket trial, SG led to a 30% reduction in the target lesions in three patients and disease stabilization in 16 (out of 25) patients.^8^ Analysis of serum samples, collected 30 minutes after the end of the SG infusion, showed that only traces of the glucuronidated metabolite (SN‐38G) were detected.^8^ Data from the Phase 2 expansion cohort of this trial indicated that most of SN‐38 in serum is bound to IgG. Free SN‐38 levels were 95.3 ng/mL (2.3%) and 56.9 ng/mL (4.5%) at 30 minutes and 1 day, respectively.^9^

SG was approved for clinical use initially by the US Food and Drug Administration (FDA) on April 20, 2020 for the treatment of adult patients with unresectable locally advanced or metastatic triple negative breast cancer (mTNBC) who have received at least two prior systemic therapies, one of which had to be in the metastatic setting.^10^ The label was subsequently expanded by the FDA to include unresectable locally advanced or metastatic hormone receptor (HR)‐positive, human epidermal growth factor receptor 2 (HER2)‐negative.^11^ In the United Kingdom, the drug was approved by the Medicines & Healthcare products Regulatory Agency (MHRA) in September 8, 2021. It is indicated for (i) the treatment of unresectable locally advanced or mTNBC who have received two or more prior lines of systemic therapies, at least one of them given for unresectable locally advanced or metastatic disease; and (ii) unresectable or metastatic HR‐positive and HER2‐negative breast cancer (HR^+^/HER2^−^ BC) who have received endocrine‐based therapy, and at least two additional systemic therapies in the advanced setting.^12^

The main toxicity reported with the clinical use of SG is similar to that observed with irinotecan, including neutropenia, febrile neutropenia and diarrhea. For example, in the pivotal ASCENT Phase 3 trial (NCT02574455), the most common treatment related adverse events (TRAEs) of any grade were neutropenia (63%), diarrhea (59%) and nausea (57%) in the SG arm. The most frequent severe (grade ≥ 3) TRAEs were neutropenia (51%), leukopenia (10%), diarrhea (10%), anemia (8%), and febrile neutropenia (6%).^13^ All these TRAEs were more common in the SG arm in comparison to the chemotherapy treated group.^13^ In the same trial, women with UGT1A1*28/*28 genotype vs. those with 1/*28 and *1/*1 genotypes had higher rates of grade ≥ 3 SG‐related neutropenia, febrile neutropenia, anemia and diarrhea.^14^ Rugo *et al*.^14^ along with the drug labels advise that individuals with the UGT1A1*28/*28 genotype should be monitored closely, but they do not recommend pre‐prescription genotyping and there are no recommendations on dose modification in individuals who have reduced UGT1A1 activity. The aim of this meta‐analysis was to understand whether, and to what extent, *UGT1A1* genotype acts as determinant of toxicity with SG.

## METHODS

### Search strategy

The systematic review and the meta‐analysis were performed in accordance with Preferred Reporting Items for Systematic Reviews and Meta‐Analysis (PRISMA) 2020 guidelines,^15^ as summarized in the Online Supplementary Content (Table S1). The study protocol was registered on the International Prospective Register of Systematic Reviews (PROSPERO, ID: CRD42024598820).

**Figure 1:**
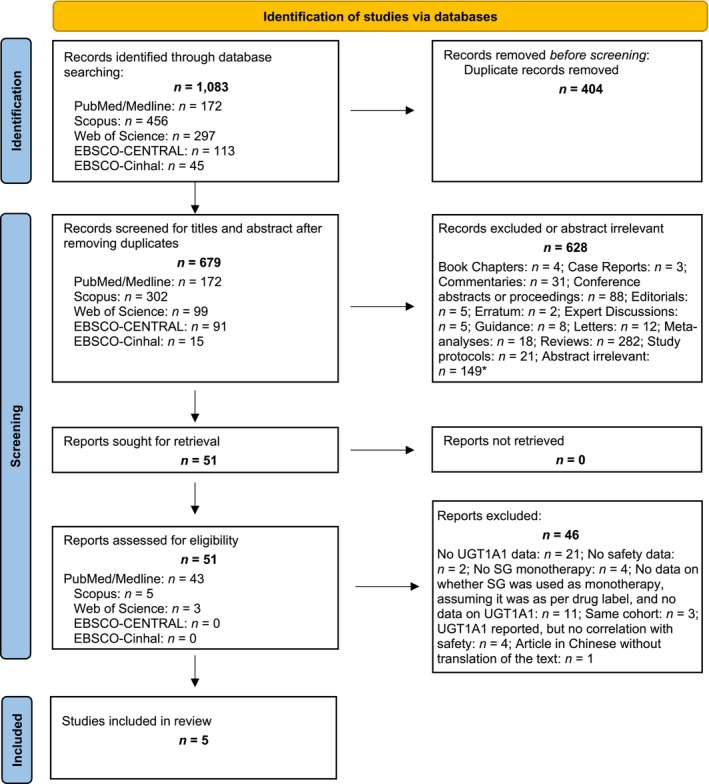
PRISMA flow diagram: Sacituzumab govitecan and toxicity. PRISMA, Preferred Reporting Items for Systematic Reviews and Meta‐Analyses. *Abstract irrelevant: Bibliometric analysis: *n* = 1; Bioanalytical assays: *n* = 5; Biomarkers/Targets (discovery and validation): *n* = 9; Database for antibodies‐drugs conjugates (ADCs): *n* = 1; Pharmacokinetics models for ADCs: *n* = 1; Drug formulation or stability: *n* = 2; Health economics: *n* = 5; Immunotherapies: *n* = 16; Improving drug development: *n* = 9; Other drugs: *n* = 65; Other Therapeutic Interventions: *n* = 4; Preclinical studies: *n* = 8; Radiotherapy: *n* = 3; Regulatory issues: *n* = 3; Sacituzumab govitecan in combination: *n* = 17.

The following databases were searched on September 29, 2024: PubMed/MEDLINE, Scopus, Web of Science, The Cochrane CENTRAL Library, and CINAHL Plus (**Figure** **1**). The search strategy, including MeSH terms, was based on the following components: ((sacituzumab govitecan‐hziy) OR (sacituzumab govitecan)) AND ((safety) OR (toxicity) OR (adverse events)) (details available in Table S2 in the Online Supplementary Content). To identify further eligible articles, lists of references from the identified studies and previous systematic reviews were hand‐searched. Additional trial results were searched in the ClinicalTrials.gov database. No restrictions or limitations (e.g., by publication date or language) were imposed on the searches.

### Study selection

Both observational (e.g., retrospective or prospective cohort and case–control studies) and interventional (e.g., randomized and non‐randomized phase 1, 2 and 3 clinical trials) studies of the association between *UGT1A1* genotype and toxicity to SG were considered eligible for inclusion in the systematic review and meta‐analysis.

One reviewer (CDR) screened the titles and abstracts of all retrieved bibliographic records according to eligibility. In addition, a second reviewer (SP) independently screened 20% of the records to check for consistency.^16^ Both reviewers (CDR and SP) were independently involved in the full‐text screening to assess the eligibility of the original articles selected via Title‐Abstract screening (**Figure** **1**). Discrepancies were resolved through discussion or by involving a third reviewer (MP) for adjudication. If an abstract was unavailable, the full text was obtained unless the article could be confidently excluded based on its title alone. In cases where there was uncertainty regarding the eligibility of a study, we proceeded to the full‐text screening stage to minimize the risk of erroneously excluding relevant studies.

For the title and abstract screening we used the following exclusion criteria: (i) Review articles; (ii) Editorials and letters without data; (iii) Expert opinions and discussions; (iv) Comments and commentaries; (v) Conference abstracts and proceedings; (vi) Case reports; (vii) Other publications (e.g., book chapters, study protocols, guidance, erratum); (viii) Pre‐clinical studies; (ix) Non‐relevant articles (e.g., articles investigating other drugs, or SG in combination).

For the full‐text screening, we used the following exclusion criteria: (i) No safety data; (ii) No *UGT1A1* genotyping data; (iii) Same cohort of patients with an already included study (please refer to the next section); (iv) SG not in monotherapy.

We excluded non‐English studies that have no translated text together with any studies from which data could not be extracted.

### Data extraction: Selection and coding

Data were added to standardized Excel files. Data included bibliographic details (first author, year of publication and PubMed/MEDLINE identifier) as well as data on the following characteristics: study design, ClinicalTrials.gov identification code, dose of SG, tumor type, length of follow‐up, overall safety population sample size (*n*), number (*n*) of patients per genotype, primary outcomes (number of patients with a specific adverse event per genotype), and other outcomes. In cases where categorical variables were presented as proportions (%), we calculated the exact number of events.

We included the following safety clinical outcomes in the analysis: any‐grade neutropenia, grade ≥ 3 neutropenia, febrile neutropenia, any‐grade anemia, grade ≥ 3 anemia, any‐grade diarrhea, and grade ≥3 diarrhea reported in the primary papers. Odd ratios (OR) with 95% confidence intervals (95% CI) were generated (dichotomous outcomes). In addition, we also evaluated the rate of dose reduction, dose/treatment interruption, and treatment discontinuation in trial participants with different genotypes. We included all clinical safety outcomes reported in the primary papers to evaluate the incidence of different toxicities in response to SG regardless of the *UGT1A1* genotype. In cases where multiple studies analyzed the same dataset for the specific exposure‐outcome combination, preference was given to publications with shorter follow‐up time and/or reporting data from a larger dataset. Patients diagnosed with advanced epithelial tumors (any type) receiving SG as a single agent and genotyped for *UGT1A1* (*28, *6 and any other variants leading to reduced enzymatic expression or activity) were included in the analysis. Among patients treated with SG, carriers of the wild‐type UGT1A1*1/*1 genotype were considered as the comparator arm.

### Risk of bias (quality) assessment

We evaluated the quality of each included study using the checklist provided by the STrengthening the Reporting of Pharmacogenetic Studies (STROPS) guideline.^17^ One reviewer (CDR) performed the assessment of risk of bias, whereas a second reviewer (IGA) independently checked for the accuracy of the evaluation. This checklist covers domains such as selection, performance, attrition, reporting, and other factors, described in the Cochrane risk of bias tool as well as those specific to pharmacogenetic studies (selecting genes/SNPs for genotyping, sample size, study design, genotype reliability, handling of missing data, population stratification, Hardy–Weinberg equilibrium, mode of inheritance, outcome selection and definition, and treatment adherence). All relevant studies were included in the meta‐analysis, although sensitivity analyses to evaluate the impact of including studies of varying quality on the pooled effect estimates could not be performed due to the limited number of studies.

### Strategy for data synthesis

We combined effect estimates for each genotype on each outcome, distinguishing between heterozygotes and homozygotes in comparison to wild‐type. We conducted meta‐analyses using R, generating ORs with 95% CIs. Forest plots were generated for each exposure‐outcome combination. We assessed the magnitude of inconsistency in study results through visual inspection of forest plots and consideration of the *I* ^2^ statistic, categorizing heterogeneity as low (*I* ^2^ < 30%), moderate (30–70%), or high (*I* ^2^ > 70%). Potential sources of heterogeneity were explored during subgroup analyses. However, analyses based on tumor type were not performed due to the limited number of studies and lack of biological rationale. Due to the limited number of clinical trials included in the analysis, the evaluation of funnel plot asymmetry to assess publication bias was not performed.

The strength of the body of evidence and the quality and strength of recommendations were assessed according to the GRADE (Grading of Recommendations, Assessment, Development and Evaluations) criteria,^18^ as shown in the Online Supplementary Content (**Table** **S3**).

### Ethics statement

The study did not require ethical approval from an institutional review board, since it is based on a quantitative analysis of publicly available data. All clinical studies included in the analysis were conducted in accordance with the Declaration of Helsinki.

## RESULTS

### Study selection

Based on the initial search results, 679 titles and abstracts were examined after the removal of duplicates (**Figure** **1**); 628 reports were rejected because they did not meet the inclusion criteria or were irrelevant to our study focus. Forty‐six of the remaining 51 full‐text articles assessed for eligibility did not meet the inclusion criteria. Checking the reference lists of identified articles and documents did not produce any additional results. Consequently, 5 publications referring to 4 clinical trials were included in the systematic review and were used for the quantitative analysis.^14^, ^19^, ^20^, ^21^, ^22^ Seven other papers^9^, ^23^, ^24^, ^25^, ^26^, ^27^, ^28^ were excluded despite appearing to meet the inclusion criteria – the reasons for this are detailed in the Online Supplementary Content (**Supplementary Results**).

Based on the modified Oxford Centre for Evidence‐based Medicine for ratings of individual studies, all pooled estimates received quality ratings of 3. Despite the fact that data were extracted from interventional (e.g., randomized and non‐randomized phase 1/2, 2 and 3 clinical trials) clinical trials, the pharmacogenetic analyses were performed retrospectively (Online Supplementary Content, **Table** **S3**).

### Study characteristics

The main characteristics of the clinical trials included in the analysis are shown in **Table** **1**. The 5 included publications were derived from 4 different clinical trials, with a total of 999 genotyped participants. The two publications on metastatic urothelial cancer (mUC) reported data from the same clinical trial (TROPHY‐U‐01, NCT03547973). However, only the first publication included data on severe (≥ 3 grade) neutropenia in a cohort of 105 patients.^20^ The most recent publication reported data on any‐grade neutropenia (and other any‐grade toxicities) in 106 patients.^21^ This publication was also used to extract data for all the other primary outcomes (any‐grade anemia and diarrhea).

**Table 1:** Main characteristics of the studies selected for the metanalysis, including study design, cancer type, median follow up, SG dose, and number of genotyped patients

PMID	First author	Year of publication	Trial name (Clinicaltrials.gov ID)	Study design	Cancer	Median Follow up (months)	SG Dose (mg/mL)	Overall safety population (*n*)	Genotyped subjects (*n*)
33741442	Bardia A	2021	IMMU‐132‐01 (NCT01631552)	Phase 1/2, non‐randomized, open label, multicenter	Advanced epithelial tumors (relapsed or refractory)	8.97 (0.26–55.72)	8 and 10 (used in the meta‐analysis)	495	403
33929895^a^	Tagawa ST	2021	TROPHY‐U‐01/IMMU‐132‐06 (NCT03547973^a^)	*Cohort 1*: Phase 2, Non‐randomized, open label, multicenter	Metastatic UC (relapsed or refractory)	9.1 (0–19.9)	10	113	105
38244927^a^	Loriot Y	2024				10.5 (0.3–40.9)	10	113	106
36038616	Rugo HS	2022	ASCENT/IMMU‐132‐05 (NCT02574455)	Phase 3, randomized, open label, multicenter	mTNBC (relapsed or refractory)	NR	10	SG group: 258	243
37633306	Rugo HS	2023	TROPiCS‐02/IMMU‐132‐09 (NCT03901339)	Phase 3, randomized, open label, multicenter	HR^+^/HER2^−^ mBC (relapsed or refractory)	SG group: 13.8 (8.3–19.8)	10	SG group: 258	247

The total number of subjects genotyped and included in the primary outcome analyses was 998–999.

^a^ These studies report data from the same clinical trial (NCT03547973). Tagawa *et al*.,^20^ was used to extract data on severe (≥ 3 grade) neutropenia, whereas Loriot *et al*.,^21^ was used to extract data on any‐grade neutropenia since it included a larger number of patients (106 vs. 105). For all the other primary outcomes, data were extracted from Loriot *et al*.^21^

All these studies enrolled patients with different advanced epithelial tumors, including mUC, mTNBC, and HR^+^/HER2^−^ mBC. All studies were interventional trials, multicenter, and carried out without masking of treatment. They were performed at different stages of clinical development; that is, one phase 1/2 (IMMU‐132‐01),^19^ one phase 2 (TROPHY‐U‐01),^20^, ^21^ and two Phase 3 (ASCENT and TROPiCS‐02)^14^, ^22^ clinical trials. In all studies, SG was used as a single agent at 10 mg/kg, except in the IMMU‐132‐01 phase 1/2 study (NCT01631552) where genotyped subjects were treated either with 8 mg/kg or 10 mg/kg.^19^

In **Table** **2**, we report the incidence of the main toxicities associated with SG treatment. On average, the most frequent toxicities associated with SG at 10 mg/kg were neutropenia (55%), diarrhea (59%) and anemia (35%). However, subjects frequently developed nausea (59%), vomiting (29%), alopecia (44%) and fatigue (45%). Data from the ASCENT and TROPiCS‐02 Phase 3 trials show that the rate of these toxicities was higher in comparison to chemotherapy.^14^, ^22^

**Table 2:** Incidence of the main toxicities observed in the selected clinical trials

Trial Name (Clinicaltrials.gov ID)/Publication	Treatment	Neutropenia (Any grade) %	Neutropenia (≥ 3 grade) %	Anemia (Any grade) %	Anemia (≥ 3 grade) %	Diarrhea (Any grade) %	Diarrhea (≥ 3 grade) %	Nausea (Any grade) %	Nausea (≥ 3 grade) %	Vomiting (Any grade) %	Vomiting (≥ 3 grade) %	Alopecia (Any grade) %	Alopecia (≥ 3 grade) %	Fatigue (Any grade) %	Fatigue (≥ 3 grade) %
IMMU‐132‐01 (NCT01631552)/Bardia A *et al*., 2021^19^	SG: 8–18 mg/kg	57.8	42.4	39.4	10.3	56.2	7.9	62.6	3.6	38.6	2.8	40.0	0	48.3	6.3
	SG: 8 mg/kg	42	NR	31	NR	52	NR	58	NR	37	NR	51	NR	51	NR
	SG: 10 mg/kg	38	NR	36	NR	57	NR	64	NR	39	NR	39	NR	47	NR
TROPHY‐U‐01/IMMU‐132‐06 (NCT03547973) / Loriot Y *et al*., 2024^21^	SG: 10 mg/kg	47	NR	34	NR	65	NR	60	NR	30	NR	47	NR	52	NR
ASCENT/IMMU‐132‐05 (NCT02574455) /Rugo HS *et al*., 2022^14^	SG: 10 mg/kg	63	51	34	8	59	10	57	3	29	1	46	0	45	3
	Chemotherapy	43	33	24	5	12	0.4	26	0.4	10	0.4	16	0	30	5.4
TROPiCS‐02 /IMMU‐132‐09 (NCT03901339)/Rugo HS *et al*., 2023^22^	SG: 10 mg/kg	70	51	34	6	57	9	55	1	19	0	46	0	38	6
	Chemotherapy	54	38	25	3	17	1	31	3	12	2	16	0	29	3

Main toxicities were selected referring to adverse events of all grades reported in >30% of SG treated subjects in the Phase 1/2 clinical trial IMMU‐132‐01.^19^ Data refers to the overall safety population (OSP, *N* = 495), including subjects treated with SG at 8–18 mg/kg. Febrile neutropenia of any grade occurred in 5.5% of the OSP, and in 5.2% of the cases was reported as severe (≥ grade 3). In this trial, 81 subjects were treated at 8 mg/mL and 402 at 10 mg/mL. Only the rate of treatment‐related adverse events was reported for these subgroups, as shown in the Table (lines 2 and 3). *UGT1A1* genotyping was performed only on subjects treated with 8–10 mg/mL. Toxicity (any grade and severe ≥ grade 3) was reported for all genotyped subjects (403/483) and data were used in the meta‐analysis.^19^

Two publications reported data on metastatic urothelial cancer (mUC) from the same clinical trial (TROPHY‐U‐01, NCT03547973).^20^, ^21^ However, only the first publication included data on severe (≥ 3 grade) neutropenia in a cohort of 105 patients.^20^ Data included in the table for this trial were extracted from the most recent publication that reported data on any grade toxicities in 106 patients.^21^

NR, not reported.

### Effect of genotype on adverse events

**Figure 2:**
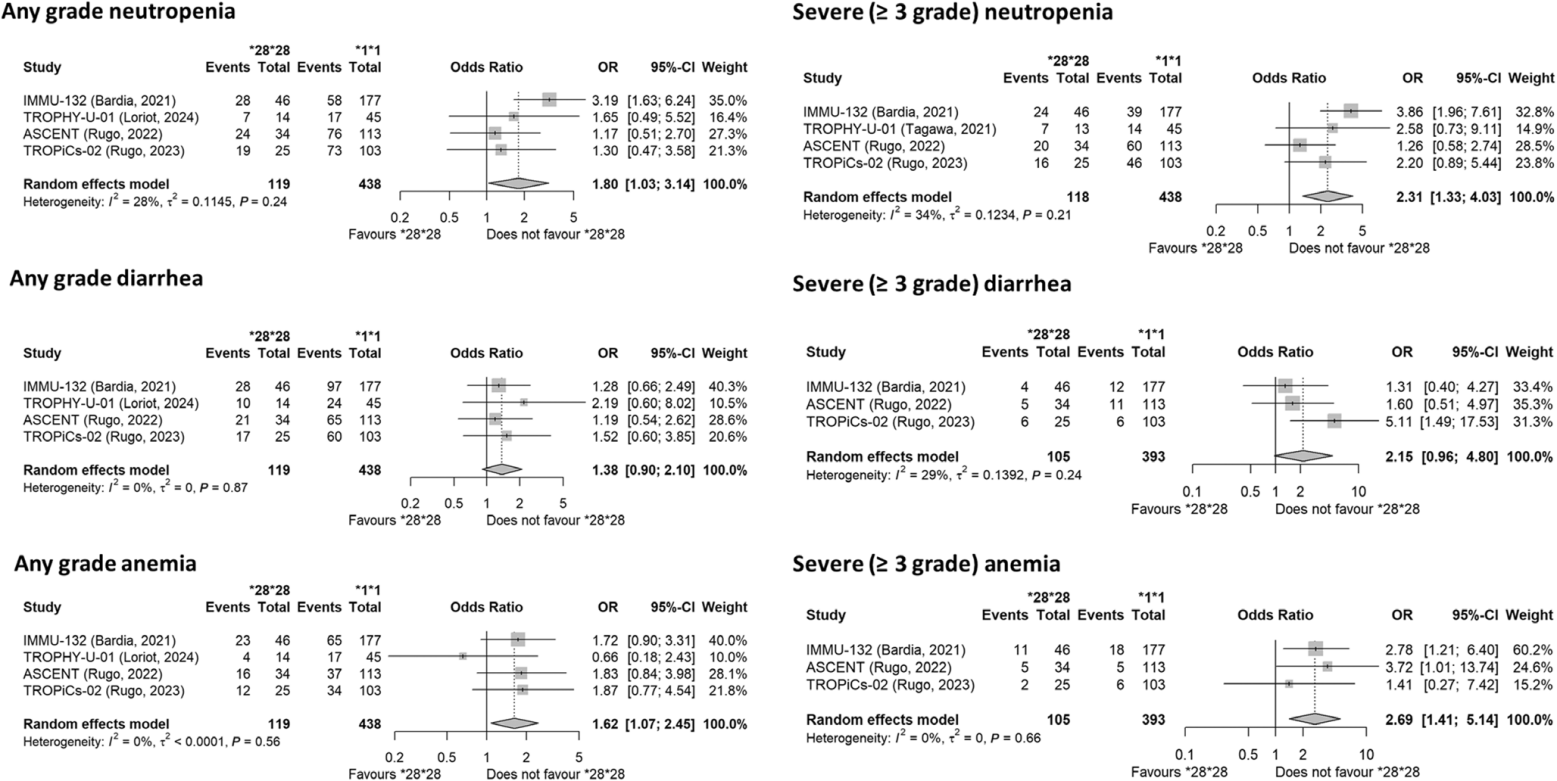
Forest plots reporting the impact of the UGT1A1*28/*28 genotype on the risk of developing different toxicities in comparison to wild‐type subjects. The effect size is calculated as odds ratio (OR) and corresponding 95% confidence interval (95% CI). Data suggest a higher risk of toxicity, particularly severe (≥ 3 grade) form of toxicity, in the homozygous subjects.

**Figure 3:**
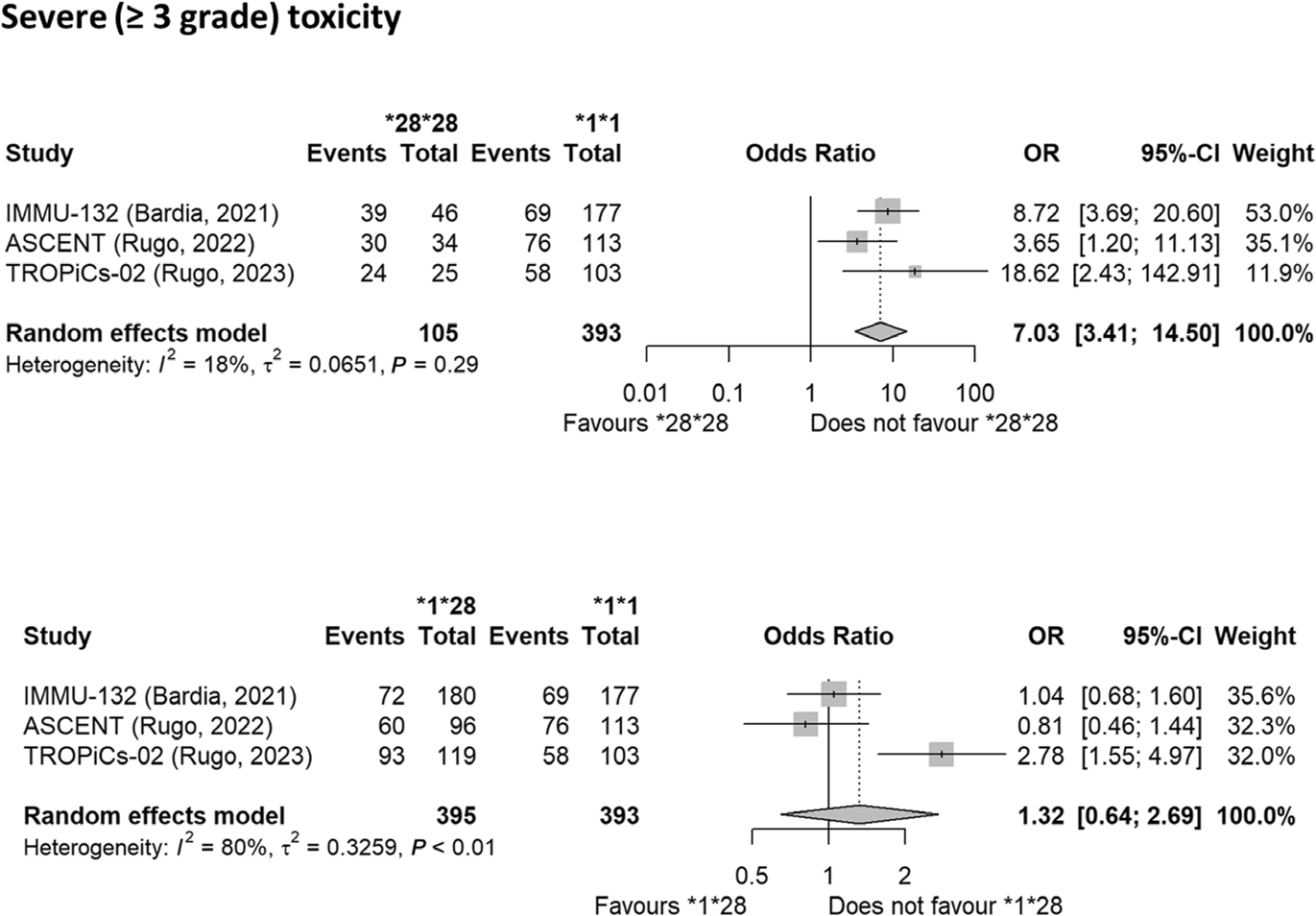
Forest plots reporting the impact of the UGT1A1*28 variant on the risk of developing severe (≥ 3 grade) toxicity in response to SGs. The effect size is calculated as odds ratio (OR) and corresponding 95% confidence interval (95% CI). Data suggest a higher risk of developing severe (≥ 3 grade) toxicity in the UGT1A1*28 homozygous subjects.

Treatment with SG in UGT1A1*28 homozygous subjects increased the risk of any‐grade neutropenia, diarrhea, and anemia (**Figure** **2**). The OR for severe (≥ 3 grade) neutropenia was 2.31 (95% CI: 1.33–4.03) in UGT1A1*28/*28 in comparison with *UGT1A1* wild‐type individuals (moderate heterogeneity, *I* ^2^ = 34%). An OR of 2.15 (95% CI: 0.96–4.80) was calculated for severe (≥ 3 grade) diarrhea (*I* ^2^ = 29%) and 2.69 (95% CI: 1.41–5.14) for severe (≥ 3 grade) anemia (*I* ^2^ = 0%) in UGT1A1*28/*28 individuals in comparison to *UGT1A1* wild‐type individuals. Notably, the OR calculated for all the severe (≥ 3 grade) forms of toxicity in UGT1A1*28 homozygous subjects treated with SG was 7.03 (95% CI: 3.41–14.50, *I* ^*2*^ = 18%) (**Figure** **3**).

**Figure 4:**
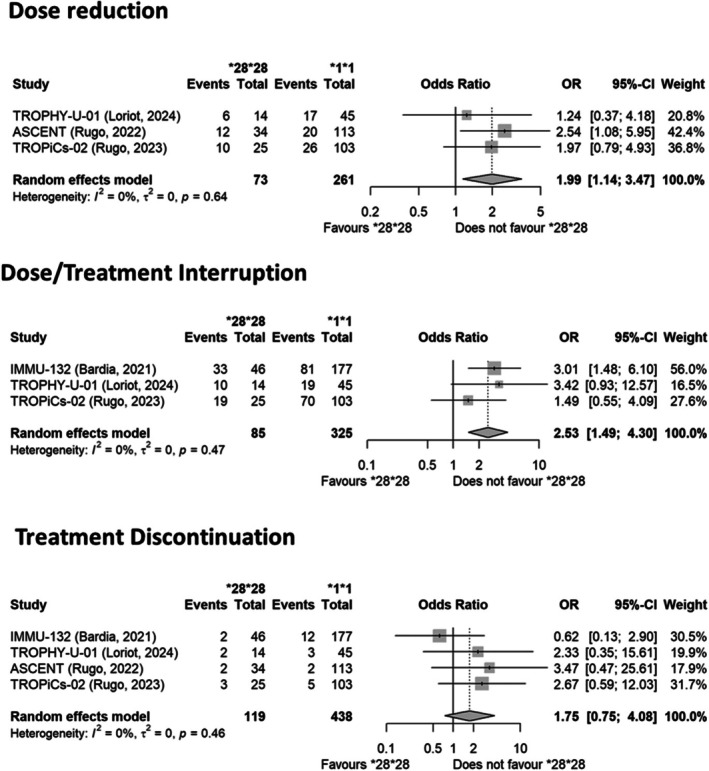
Forest plots reporting the impact of the UGT1A1*28 variant on the risk of dose modification/suspension/interruption in subjects treated with SGs. The effect size is calculated as odds ratio (OR) and the corresponding 95% confidence interval (95% CI). Data suggest an increased risk of dose reduction and dose/treatment interruption in carriers of the UGT1A1*28/*28 genotype vs. wild‐type subjects.

In addition, reduced adherence to the treatment schedule was observed in subjects carrying the UGT1A1*28/*28 genotype (**Figure** **4**). In particular, the ORs (95% CI) were 1.99 (1.14–3.47, *I* ^*2*^ = 0%) and 2.53 (1.49–4.30, *I* ^*2*^ = 0%) for dose reduction and dose/treatment interruption, respectively, in UGT1A1*28 homozygous subjects vs. *UGT1A1* wild type individuals. There was also a trend toward an increased rate of treatment discontinuation in UGT1A1*28 homozygous subjects vs. *UGT1A1* wild‐type individuals (**Figure** **4**).

We did not find a significant increase in the risk of neutropenia, diarrhea, and anemia (any grade or severe) in UGT1A1*28 heterozygous patients treated with SG as a single agent in comparison to *UGT1A1* wild‐type patients (Online Supplementary Content, **Figure** **S1**). Similarly, the OR for combined severe toxicities was not significantly increased in UGT1A1*28 heterozygous subjects treated with SG (**Figure** **3**). Cases of febrile neutropenia were reported as severe (≥ 3 grade) toxicity, and data on febrile neutropenia were available only for metastatic breast cancer^14^, ^22^ – this showed a trend toward an increased risk of severe febrile neutropenia in UGT1A1*28 homozygous subjects in comparison to *UGT1A1* wild‐type subjects (OR 2.64, 95% CI: 0.24–28.86, *I* ^2^ = 71) (Online Supplementary Content, **Figure** **S2**). The risk of dose reduction, dose/treatment interruption, and treatment discontinuation did not vary in UGT1A1*28 heterozygous subjects in comparison to wild‐type individuals (Online Supplementary Content, **Figure** **S3**).

The available data did not provide sufficient results to draw conclusions about the interaction between toxicity to SG and UGT1A1*6.

### Assessment for risk of bias

We evaluated the quality of each included study using the checklist provided by the STrengthening the Reporting Of Pharmacogenetic Studies (STROPS) guideline.^17^ As summarized in **Table** **3**, most of the criteria relevant for pharmacogenomic association studies were not met. All the data used for the meta‐analysis derived from 4 interventional clinical studies carried out at different stages of clinical development, including one phase 1/2 (IMMU‐132‐01),^19^ one phase 2 (TROPHY‐U‐01),^20^, ^21^ and two Phase 3 (ASCENT and TROPiCS‐02)^14^, ^22^ clinical trials. All studies were multicenter studies, sponsored by Gilead Science, and were carried out at different locations mostly in North America and Europe/UK without masking of the treatment. Only the ASCENT and TROPiCS‐02 Phase 3 trials were randomized and included a control arm. Subjects, both females and males, were enrolled based on specific inclusion/exclusion criteria, which were mostly based on the specific tumor type, ECOG performance status, renal and liver function, and hematological function. Interestingly, in the initial IMMU‐132‐01 phase 1/2 study^9^ and in the ASCENT Phase 3 trial undertaken in TNBC^14^, participants with Gilbert’s disease, a benign condition associated with UGT1A1*28, were excluded, whereas in the TROPiCS‐02 Phase 3 trial individuals with documented Gilbert’s syndrome were enrolled if bilirubin plasma levels were ≤ 3× upper limit of normal.^22^ The primary outcome measure in all these trials was either safety or efficacy.

**Table 3:** Evaluation of the risk of bias. The evaluation of the risk of bias for the association between safety outcomes and UGT1A1 allele variants was performed by using the STrengthening the Reporting of Pharmacogenetic Studies (STROPS) guideline

Category	Criteria #	Bardia (2021)^19^	Loriot (2024)^21^	Rugo (2022)^14^	Rugo (2023)^22^
Abstract					
Abstract	1	Probably no	Probably no	Probably no	No
Introduction					
Background/Rationale	2	No	Yes	Yes	No
	3	No	Yes	Yes	No
Objectives	4	No	No	No	No
	5	Yes	Yes	Yes	Yes
Methods					
Study design	6	Yes	Yes	Probably yes	Probably yes
Setting	7	Yes	Yes	Yes	Yes
Participants	8	Probably yes	Probably yes	Probably yes	Probably yes
	9	Yes	Yes	Yes	Yes
	10	Not applicable	Not applicable	Not applicable	Not applicable
	11	Not applicable	Not applicable	Not applicable	Not applicable
	12	Probably yes	Yes	No	No
	13	Yes	Yes	Yes	Yes
Variables	14	Probably yes	Probably yes	Probably yes	Probably yes
	15	Probably yes	Probably yes	Probably yes	Probably yes
	16	Yes	Yes	Yes	Yes
	17	No	Yes	No	No
	18	No	No	No	No
	19	Probably yes	Probably yes	Probably yes	Probably yes
Data sources/measurement	20	Probably yes	Probably yes	Probably yes	Probably yes
	21	No	No	No	Probably no
	22	No	No	No	No
	23	No	No	No	No
	24	Probably yes	Probably yes	Probably yes	Probably yes
Study size	25	No	No	No	No
	26	Not applicable	Not applicable	Not applicable	Not applicable
Statistical methods	27	Probably no	Probably no	Probably No	Probably no
	28	No	No	No	No
	29	No	No	No	No
	30	No	No	No	No
	31	No	No	No	No
	32	No	No	No	No
	33	Probably yes	Probably yes	Probably yes	Probably yes
Results					
Participants	34	Probably no	Probably no	Probably no	Probably no
SNPs	35	Not applicable	Not applicable	Not applicable	Not applicable
Descriptive data	36	No	No	No	No
	37	Yes	Yes	No	Yes
	38	No	No	No	No
	39	No	No	No	No
Outcome data	40a	Probably yes	Probably yes	Probably yes	Probably yes
	41	No	No	No	No
Main results	42	Probably yes	Probably yes	Probably yes	Probably yes
	43	Not applicable	Not applicable	Not applicable	Not applicable
Other analyses	44	Not applicable	Not applicable	Not applicable	Not applicable
	45	Not applicable	Not applicable	Not applicable	Not applicable
	46	Yes	Yes	Yes	Yes
Discussion					
Key results	47	Probably yes	Probably yes	Probably yes	Probably yes
Limitations	48	No	No	No	No
Interpretation	49	Probably yes	Probably yes	Probably yes	Probably no
Generalizability	50	Not applicable	Not applicable	Not applicable	Not applicable
Other information					
Study registration	51	Yes	Yes	Yes	Yes
Ethical approval	52	Yes	Yes	Yes	Yes
Funding	53	Yes	Yes	Yes	Yes
Database	54	Yes	Yes	Yes	Yes

None of the trials were designed to assess the pharmacogenetic association between SG toxicity and *UGT1A1* genetic variability. Therefore, all studies lacked *a priori* statistical analysis to establish sample size and power for the detection of a pharmacogenetic association between UGT1A1*28 and different SG toxicities. Nevertheless, *UGT1A1* testing was required for all subjects enrolled in these trials, although treatment was not stratified based on the *UGT1A1* status. Potential confounders, such as low absolute neutrophil count and hemoglobin at baseline due to previous therapies, impaired renal and liver function, and previous toxicity to irinotecan, were controlled by excluding participants at enrolment. Considering that all these studies were not designed for the evaluation of the pharmacogenetic association, it is not surprising that the methodology for genotyping is poorly described. Only one Phase 3 trial clearly stated that genotyping was performed centrally at Covance Genomics Lab (LabCorp, Burlington, NC).^22^ Genotyping was not done or was missing in a small percentage of subjects; this was simply reported without any description of how missing data were addressed. Hardy–Weinberg equilibrium was not considered, although the frequency of each *UGT1A1* genotype was provided and was in line with the expected population frequency. Population stratification was not assessed. All the studies enrolled mostly White individuals, from 67 to 81%. Methods used to assess and correct for relatedness among subjects were not reported, although most likely these individuals were not related. Finally, the STROPS guidelines do not account for factors such as word count limitations, which may restrict details reported in abstracts. Detailed information on each of the characteristics included in the STROPS guidelines is reported in the Online Supplementary Content (**Table** **S4**).

## DISCUSSION

Despite SG being an ADC designed to maximize tumor delivery of SN‐38, the serious adverse reactions observed with SG resemble the toxic effects of irinotecan (the pro‐drug of SN‐38), in particular severe neutropenia, diarrhea, and anemia.^19^, ^21^, ^22^, ^24^ This indicates that systemic exposure to SN‐38 is at a high enough level to cause systemic toxicity. A recent meta‐analysis of 7 clinical studies in breast cancer reported that the most frequent toxicities associated with SG were neutropenia [Absolute Risk (AR) of 70%, with 95% CI of 64–76%], nausea (AR of 62%, with 95% CI of 55–68%), diarrhea (AR of 54%, with 95% CI of 47–60%) and anemia (AR of 51%, with 95% CI of 38–65%).^29^ Data from ASCENT and TROPiCS‐02 Phase 3 trials found that the rate of these toxicities was higher in comparison to the treatment of physician’s choice.^14^, ^22^ Furthermore, meta‐analyses confirmed that a higher risk of toxicity was observed in subjects treated with SG in comparison to those exposed to chemotherapy.^29^, ^30^

Toxicity may be worsened in those with deficient activity of UGT1A1, the main detoxification enzyme. This is confirmed in our current systematic review and meta‐analysis, which demonstrates that individuals with the UGT1A*28/*28 genotype were at an increased risk of toxicity. The OR for severe (≥ 3 grade) neutropenia, anemia, and diarrhea was > 2 in UGT1A1*28/*28 individuals in comparison to individuals who were *UGT1A1* wild‐type, and the OR for combined severe toxicity was around 7. In addition, the risk of treatment modification and interruption was doubled in subjects with the UGT1A1*28/*28 genotype in comparison to wild‐type, indicating lower adherence to the treatment schedule in this subgroup. Data are consistent with both the IMMU‐132‐01 Phase 1/2 clinical trial (NCT01631552)^19^ and the ASCENT Phase 3 trial (NCT02574455)^14^ which demonstrated an increased incidence of serious adverse events in UGT1A1*28 homozygous subjects in comparison to wild‐type individuals (87.6% vs. 53.3%; SAE relative risk = 1.55, 95% CI: 1.46–1.87, *I* ^2^ = 0%).^31^ It is important to note that although both the ASCENT and TROPiCS‐02 Phase 3 clinical trials showed improved health‐related quality of life in patients treated with SG compared to chemotherapy, this was not stratified based on UGT1A1*28 genotype.^32^, ^33^

Our systematic review and meta‐analysis has focused on UGT1A1*28 because of data availability. A limitation of our analysis is that we could not evaluate other variants in *UGT1A1* that may be important in different ethnic populations. In EVER‐132‐001 (NCT04454437), a multicenter, single arm, phase 2b study in Chinese subjects with mTNBC who failed ≥ 2 prior chemotherapy regimens, there was an increased risk of toxicity in carriers of the *6 variant, also categorized as *UGT1A1* poor metabolizers.^25^ UGT1A1*6/*6 homozygous subjects had a higher incidence of grade ≥ 3 neutropenia (100%) and anemia (66.7%) in comparison with wild‐type *UGT1A1* subjects (52.6% and 10.5%, respectively) – this data was only mentioned in the discussion.^25^ Similarly, in the EVER‐132‐002 Phase 3 clinical trial (NCT04639986), which became available outside our data lock point, there was an increased rate of toxicity and severe toxicity with SG in UGT1A1*6 (40 subjects) and UGT1A1*28 (18 subjects) heterozygous individuals with HR^+^/HER2^−^ breast tumors in comparison to 77 wild‐type individuals.^34^

The Dutch Pharmacogenetics Working Group^3^ recommends dose reductions in *UGT1A1* poor metabolizers being treated with irinotecan, but this guideline does not cover SG. In the pivotal ASCENT trial,^14^ the authors noted a higher rate of grade ≥ 3 SG‐related neutropenia, febrile neutropenia, anemia, and diarrhea in women with the UGT1A1*28/*28 genotype and advised that individuals with the UGT1A1*28/*28 genotype should be monitored closely. The FDA and MHRA drug labels also acknowledge the role of the *UGT1A1* polymorphisms, but there are no recommendations on dose modification in individuals who have reduced UGT1A1 activity. However, these regulatory agencies advise that the dose of SG should be reduced by 25% at the first occurrence of severe hematological or other types of toxicities. In addition, both recommend to “*withhold or permanently discontinue TRODELVY*  (SG) *based on clinical assessment of the onset, duration and severity of the observed adverse reactions in patients with evidence of acute early‐onset or unusually severe adverse reactions, which may indicate reduced UGT1A1 enzyme activity*”.^10^, ^12^

For irinotecan itself, the Dutch Pharmacogenetics Working Group guideline^3^ recommends a 30% dose reduction in UGT1A1*28 homozygous subjects (but not in heterozygous), with the option to increase the dose (guided by the neutrophil count) if the initial dose is tolerated. This dose recommendation is based on evaluation of the pharmacokinetics of SN‐38, the active metabolite of irinotecan. This showed that the AUC of SN‐38 increased by 18‐159% and the clearance decreased by 61% in UGT1A1*28 homozygous subjects compared with wild‐type subjects, equating to a dose reduction to 69% (range 48–92%) (please see appendix in the guideline).^3^ Thus, a similar approach could be considered in patients treated with SG, with a pre‐emptive 25% dose reduction in UGT1A1*28 homozygous subjects. The choice of 25% dose reduction is pragmatic because this level of dose reduction is already in the label for those who develop toxicity. Unfortunately, we are not aware of any pharmacokinetic evaluation of SN‐38 in UGT1A1*28 homozygous compared with wild‐type subjects. However, our data clearly show that at the current dosing regimen for SG, there is a 7‐fold increased risk of severe toxicity in UGT1A1*28 homozygous subjects. Clinicians and patients may worry that a reduction in dose will lead to a reduction in efficacy. In this regard, recent evidence suggests that the recommended/standard dose of SG can be reduced without significant reduction in drug exposure and therefore its efficacy.^35^ Concerns have also been expressed about 5‐fluorouracil dose reduction in individuals with loss‐of‐function variants in the *DPYD* gene, but this is now standard practice in most European countries. In addition, a recent sub‐study of the PREPARE trial from the Italian oncology recruiting sites showed that *DPYD* and *UGT1A1* variant carriers had a 90% lower risk of serious toxicity, had a 3‐fold lower rate of hospitalization and cost less ($4,159/patients in the control arm, and $26 in the intervention arm).^36^ Furthermore, this analysis showed that the cumulative dose received in patients with the 4 *DPYD* variants was actually greater than in “wild‐type” individuals because the higher rate of toxicity in the latter group led to either empiric dose reduction or drug discontinuation.

An alternative approach to dose reduction in UGT1A1*28 homozygous subjects, in the context of mTNBC, may be to use trastuzumab‐deruxtecan instead, which has been shown to have comparable results to SG as a second or later line of treatment.^37^, ^38^ Indeed, similar efficacy of trastuzumab‐deruxtecan in comparison to SG has also been recently confirmed by a network meta‐analysis in metastatic breast cancer including TNBC and HR^+^/HER2^−^ subjects.^39^ Thus, trastuzumab‐deruxtecan may be a valid alternative in patients with reduced UGT1A1 function, but both its other adverse effects (for example pneumonitis) and cost would need to be considered.

In conclusion, pre‐treatment *UGT1A1* genotyping should be considered to optimize SG clinical use, identifying individuals that may benefit from personalized dosing (for example, a 25% dose reduction from the start), closer monitoring, or alternative treatments (trastuzumab‐deruxtecan) to reduce toxicity.

## FUNDING

This work was supported by the NHS Network of Excellence in Pharmacogenomics and Medicines Optimisation. It was also supported by the UKRI/ESRC “The organisation and diffusion of translational research: Can cardiovascular medicine learn from oncology? Case studies of pharmacogenomics in the NHS” (Grant #: ESRC – ES/W011484/1) and by the Innovate UK ‘Centre for Excellence in Regulatory Science and Innovation in Pharmacogenomics’ (Project # 10140264).

## CONFLICTS OF INTEREST

M.P. currently receives partnership funding for the following: MRC Clinical Pharmacology Training Scheme (co‐funded by MRC and Roche, UCB, Eli Lilly and Novartis). He has developed an HLA genotyping panel with MC Diagnostics, but does not benefit financially from this. He is part of the IMI Consortium ARDAT (www.ardat.org). None of these funding sources will be used for this work. C.P. reports consulting or advisory roles for, Daiichi Sankyo, Exact Sciences, Gilead, MSD, Menarini/Stemline Novartis, Pfizer and Seagen; research funding from, Pfizer, Daiichi Sankyo, Exact Sciences, Gilead and Seagen and travel support from, AZ, Gilead, Novartis, Pfizer and Roche. All other authors declared no competing interests for this work.

## AUTHOR CONTRIBUTIONS

C.D.R., C.P., and M.P. wrote the manuscript; C.D.R., I.G.A., C.P., and M.P. designed the research; C.D.R. and S.P. performed the research; C.D.R. and I.G.A. analyzed the data.

## Supporting information


Data S1
