# Supplementary material for: UGT1A1 and Sacituzumab Govitecan Toxicity: A Systematic Review and Meta‐Analysis
Source: Clin Pharmacol Ther. 2025 Sep 11;119(1):63–73. doi: 10.1002/cpt.70060 (PMC12746514; doi:10.1002/cpt.70060)
Supplement: Supplementary file 1 — Data S1 [file CPT-119-63-s001.docx]

**SUPPLEMENTARY MATERIAL**

**UGT1A1 and sacituzumab govitecan toxicity: a systematic review and meta-analysis.**

Cinzia Dello Russo^1,2^ (<https://orcid.org/0000-0002-2538-3832>), Innocent Gerald Asiimwe^3^ (<https://orcid.org/0000-0002-1196-1822>), Sudeep Pushpakom^1,4^ (<https://orcid.org/0000-0002-6682-4235>), Carlo Palmieri^5,6^ (<https://orcid.org/0000-0001-9496-2718>), Munir Pirmohamed^1,4^ (<https://orcid.org/0000-0002-7534-7266>).

^1^Department of Pharmacology and Therapeutics, Institute of Systems, Molecular and Integrative Biology, University of Liverpool, Liverpool, UK.

^2^Department of Healthcare Surveillance and Bioethics, Section of Pharmacology, Università Cattolica del Sacro Cuore - Fondazione Policlinico Universitario A. Gemelli, IRCCS, Rome, Italy.

^3^Department of Health Data Science, Institute of Population Health, University of Liverpool, Liverpool, UK.

^4^The Wolfson Centre for Personalised Medicine, MRC Centre for Drug Safety Science, University of Liverpool, Liverpool, UK.

^5^Department of Molecular and Clinical Cancer Medicine, Institute of Systems, Molecular and Integrative Biology, University of Liverpool, Liverpool UK

^6^The Clatterbridge Cancer Centre NHS Foundation Trust, Liverpool, UK.

**Supplementary results**

As shown in figure 1, 679 titles and abstracts were examined after removal of duplicates, from which 5 publications referring to 4 clinical trials were included in the systematic review. Seven other papers^1-7^ were excluded despite appearing to meet the inclusion criteria. Three papers were excluded because they reported interim analyses on the same cohorts, although on a smaller number of individuals^1-3^. In a phase 2b study that enrolled Chinese subjects with mTNBC (NCT04454437), both *UGT1A1*6* and **28* variants were genotyped, although safety data per genotype are partially reported in the discussion^4^. ASCENT-J02 (NCT05101096) was a phase 1/2 study carried out in Japanese individuals with advanced solid tumors. Preliminary data from this trial have been published recently, including general safety data based on *UGT1A1* genotype, without relevant differences detected in both phases. However, the dose-expansion mTNBC cohort (phase 2), included 36 subjects of whom only one **28* homozygous and three heterozygous (**6* or **28*)^5^. We also excluded two retrospective studies. The first one included as safety outcome the hazard ratios for treatment discontinuation. Moreover, 76% of individuals *UGT1A1*28* homozygous were started at lower dose^6^. The study NCT02284581 is retrospective observational study including subjects with mTNBC. Only 17 (28%) individuals were genotyped, and data were briefly discussed^7^.

**Supplementary Tables**

**Table S1. Preferred Reporting Items for Systematic Reviews and Meta-Analyses: The PRISMA Statement^8^.**

| **Section and Topic** | **Item #** | **Checklist item** | **Location where item is reported** |
| --- | --- | --- | --- |
| **TITLE** | | |  |
| Title | 1 | Identify the report as a systematic review. | Title page |
| **ABSTRACT** | | |  |
| Abstract | 2 | See the PRISMA 2020 for Abstracts checklist. | Abstract |
| **INTRODUCTION** | | |  |
| Rationale | 3 | Describe the rationale for the review in the context of existing knowledge. | Introduction, Paragraphs 1-6 |
| Objectives | 4 | Provide an explicit statement of the objective(s) or question(s) the review addresses. | Introduction, Paragraph 7 |
| **METHODS** | | |  |
| Eligibility criteria | 5 | Specify the inclusion and exclusion criteria for the review and how studies were grouped for the syntheses. | Methods, Study Selection |
| Information sources | 6 | Specify all databases, registers, websites, organisations, reference lists and other sources searched or consulted to identify studies. Specify the date when each source was last searched or consulted. | Methods, Search Strategy and Online Supplementary Content, Table S2 |
| Search strategy | 7 | Present the full search strategies for all databases, registers and websites, including any filters and limits used. | Online Supplementary Content, Table S2 |
| Selection process | 8 | Specify the methods used to decide whether a study met the inclusion criteria of the review, including how many reviewers screened each record and each report retrieved, whether they worked independently, and if applicable, details of automation tools used in the process. | Methods, Study Selection |
| Data collection process | 9 | Specify the methods used to collect data from reports, including how many reviewers collected data from each report, whether they worked independently, any processes for obtaining or confirming data from study investigators, and if applicable, details of automation tools used in the process. | Methods, Data extraction: selection and coding |
| Data items | 10a | List and define all outcomes for which data were sought. Specify whether all results that were compatible with each outcome domain in each study were sought (e.g. for all measures, time points, analyses), and if not, the methods used to decide which results to collect. | Methods, Data extraction: selection and coding. |
|  | 10b | List and define all other variables for which data were sought (e.g. participant and intervention characteristics, funding sources). Describe any assumptions made about any missing or unclear information. |  |
| Study risk of bias assessment | 11 | Specify the methods used to assess risk of bias in the included studies, including details of the tool(s) used, how many reviewers assessed each study and whether they worked independently, and if applicable, details of automation tools used in the process. | Methods, Risk of bias (quality) assessment |
| Effect measures | 12 | Specify for each outcome the effect measure(s) (e.g. risk ratio, mean difference) used in the synthesis or presentation of results. | Methods, Strategy for data synthesis |
| Synthesis methods | 13a | Describe the processes used to decide which studies were eligible for each synthesis (e.g. tabulating the study intervention characteristics and comparing against the planned groups for each synthesis (item #5)). | Methods, Strategy for data synthesis |
|  | 13b | Describe any methods required to prepare the data for presentation or synthesis, such as handling of missing summary statistics, or data conversions. |  |
|  | 13c | Describe any methods used to tabulate or visually display results of individual studies and syntheses. |  |
|  | 13d | Describe any methods used to synthesize results and provide a rationale for the choice(s). If meta-analysis was performed, describe the model(s), method(s) to identify the presence and extent of statistical heterogeneity, and software package(s) used. |  |
|  | 13e | Describe any methods used to explore possible causes of heterogeneity among study results (e.g. subgroup analysis, meta-regression). |  |
|  | 13f | Describe any sensitivity analyses conducted to assess robustness of the synthesized results. |  |
| Reporting bias assessment | 14 | Describe any methods used to assess risk of bias due to missing results in a synthesis (arising from reporting biases). | Not applicable |
| Certainty assessment | 15 | Describe any methods used to assess certainty (or confidence) in the body of evidence for an outcome. | Methods, Strategy for data synthesis |
| **RESULTS** | | |  |
| Study selection | 16a | Describe the results of the search and selection process, from the number of records identified in the search to the number of studies included in the review, ideally using a flow diagram. | Results, Study selection and Figure 1. |
|  | 16b | Cite studies that might appear to meet the inclusion criteria, but which were excluded, and explain why they were excluded. | Results, Study selection |
| Study characteristics | 17 | Cite each included study and present its characteristics. | Results, Study characteristics and Table 1 |
| Risk of bias in studies | 18 | Present assessments of risk of bias for each included study. | Results, Assessment for Risk of Bias, Table 3 and Online Supplementary Content Table S4 |
| Results of individual studies | 19 | For all outcomes, present, for each study: (a) summary statistics for each group (where appropriate) and (b) an effect estimate and its precision (e.g. confidence/credible interval), ideally using structured tables or plots. | All forest plots and Table 2 |
| Results of syntheses | 20a | For each synthesis, briefly summarise the characteristics and risk of bias among contributing studies. | All forest plots |
|  | 20b | Present results of all statistical syntheses conducted. If meta-analysis was done, present for each the summary estimate and its precision (e.g. confidence/credible interval) and measures of statistical heterogeneity. If comparing groups, describe the direction of the effect. |  |
|  | 20c | Present results of all investigations of possible causes of heterogeneity among study results. |  |
|  | 20d | Present results of all sensitivity analyses conducted to assess the robustness of the synthesized results. | Not performed |
| Reporting biases | 21 | Present assessments of risk of bias due to missing results (arising from reporting biases) for each synthesis assessed. | Not applicable |
| Certainty of evidence | 22 | Present assessments of certainty (or confidence) in the body of evidence for each outcome assessed. | Results, Study selection |
| **DISCUSSION** | | |  |
| Discussion | 23a | Provide a general interpretation of the results in the context of other evidence. | Discussion, Paragraph 1 |
|  | 23b | Discuss any limitations of the evidence included in the review. | Discussion, Paragraph 3 |
|  | 23c | Discuss any limitations of the review processes used. | Discussion, Paragraph 3 |
|  | 23d | Discuss implications of the results for practice, policy, and future research. | Discussion, Paragraphs 5-7 |
| **OTHER INFORMATION** | | |  |
| Registration and protocol | 24a | Provide registration information for the review, including register name and registration number, or state that the review was not registered. | Abstract; Methods, Search strategy |
|  | 24b | Indicate where the review protocol can be accessed, or state that a protocol was not prepared. |  |
|  | 24c | Describe and explain any amendments to information provided at registration or in the protocol. |  |
| Support | 25 | Describe sources of financial or non-financial support for the review, and the role of the funders or sponsors in the review. | Acknowledgements |
| Competing interests | 26 | Declare any competing interests of review authors. | Competing Interests |
| Availability of data, code and other materials | 27 | Report which of the following are publicly available and where they can be found: template data collection forms; data extracted from included studies; data used for all analyses; analytic code; any other materials used in the review. | All data are included in the publication |

**Table. S2 Search strategy**

**Data cutoff date: 29 September 2024**

| **PubMed/MEDLINE** |
| --- |
| **Search strings:** ((Sacituzumab govitecan-hziy) OR (sacituzumab govitecan)) AND ((safety) OR (toxicity) OR (adverse events)) |
| **Details:** *(("sacituzumab"[All Fields] AND "govitecan-hziy"[All Fields]) OR ("sacituzumab govitecan"[Supplementary Concept] OR "sacituzumab govitecan"[All Fields])) AND ("toxic"[All Fields] OR "toxical"[All Fields] OR "toxically"[All Fields] OR "toxicant"[All Fields] OR "toxicant s"[All Fields] OR "toxicants"[All Fields] OR "toxicated"[All Fields] OR "toxication"[All Fields] OR "toxicities"[All Fields] OR "toxicity"[MeSH Subheading] OR "toxicity"[All Fields] OR "toxicity s"[All Fields] OR "toxics"[All Fields] OR ("safety"[MeSH Terms] OR "safety"[All Fields] OR "safeties"[All Fields]) OR (("adverse"[All Fields] OR "adversely"[All Fields] OR "adverses"[All Fields]) AND ("event"[All Fields] OR "event s"[All Fields] OR "events"[All Fields])))* |
| **Retrieved articles: 172** |
|  |
| **SCOPUS** |
| **Search strings:** (TITLE-ABS-KEY(((Sacituzumab govitecan-hziy) OR (sacituzumab govitecan)) AND ((safety) OR (toxicity) OR (adverse events)))) |
| **Retrieved articles: 456** |
| **Duplicates: 154** |
| **Not duplicates: 302** |
|  |
| **Web of Science Core Collection:** ((Sacituzumab govitecan-hziy) OR (sacituzumab govitecan)) AND ((safety) OR (toxicity) OR (adverse events)) |
| **Retrieved articles: 297** |
| **Duplicates: 198** |
| **Not duplicates: 99** |
|  |
| **EBSCO-CENTRAL:** ((Sacituzumab govitecan-hziy) OR (sacituzumab govitecan)) AND ((safety) OR (toxicity) OR (adverse events)) |
| **Retrieved articles: 113** |
| **Duplicates: 22** |
| **Not duplicates: 91** |
|  |
| **EBSCO-Cinhal:** ((Sacituzumab govitecan-hziy) OR (sacituzumab govitecan)) AND ((safety) OR (toxicity) OR (adverse events)) |
| **Retrieved articles: 45** |
| **Duplicates: 30** |
| **Not duplicates: 15** |
|  |
| **Total articles to be screened: 679** |

**Table S3. Modified Oxford Centre for Evidence-based Medicine for ratings of individual studies^9^ (**<https://jamanetwork.com/journals/jama/pages/instructions-for-authors#SecMeta-analysis>)**.**

| **Quality Rating Scheme for Studies and Other Evidence** | |
| --- | --- |
| 1 | Properly powered and conducted randomized clinical trial; systematic review with meta-analysis |
| 2 | Well-designed controlled trial without randomization; prospective comparative cohort trial |
| 3 | Case-control studies; retrospective cohort study |
| 4 | Case series with or without intervention; cross-sectional study |
| 5 | Opinion of respected authorities; case reports |

**Table S4. Evaluation of the Risk of Bias.** The evaluation of the risk of bias for the association between safety outcomes and UGT1A1 allele variants was performed by using the STrengthening the Reporting Of Pharmacogenetic Studies: Development of the STROPS guideline^10^.

| **CATEGORY** | **#** | **Criteria** | **Bardia (2021)^11^** | **Loriot (2024)^12^** | **Rugo (2022)^13^** | **Rugo (2023)^14^** |
| --- | --- | --- | --- | --- | --- | --- |
| **ABSTRACT** |  |  |  |  |  |  |
| **Abstract** | 1 | Provide in the abstract an informative and balanced summary of what was done and what was found. | **Probably No**  The abstract reports only the number of subjects with known *UGT1A1* genotype with respect to the allele **28* in the overall safety population (n = 495) and the incidence of neutropenia (all grades) per genotype. | **Probably No**  The abstract reports only the percentage of subjects with known *UGT1A1* genotype with respect to the allele **28* in the population enrolled in Cohort 1 of the study (n = 113) and the incidence of treatment-related adverse events (any grades) per genotype. | **Probably No**  The abstract reports the  safety analysis for the ASCENT trial, including the impact of *UGT1A1* genetic variability. In this regard, it is only provided the increased rate of toxic effects, such as grade ≥3 SG-related neutropenia, febrile neutropenia, anemia e diarrhoea, in *UGT1A1*28/*28* subjects in comparison to the other subgroups. Moreover, the authors advise that individuals with *UGT1A1*28/*28* genotype need to be monitored closely. They suggest that active monitoring and the routine management of adverse effects allow for the optimization of SG therapeutic exposure. | **No**  The abstract does not refer to *UGT1A1* genotyping and possibly related safety data. |
| **INTRODUCTION** |  |  |  |  |  |  |
| **Background/Rationale** | 2 | Explain the scientific background and rationale for the investigation being reported. | **No**  The scientific background or rationale for the *UGT1A1* genotyping is not explained. In addition, it is mentioned that previous data suggest lack of correlation between toxicity and *UGT1A1* genetic variability^1^. | **Yes**  The scientific background or rationale for *UGT1A1* genotyping is explained. In addition, it is reported that data from the ASCENT trial suggest increased hematological toxicities and diarrhea in subjects who are homozygous for *UGT1A1*28*. However, the authors state that the rate of discontinuation due to treatment-related AEs is low^13^. | **Yes**  The scientific background or rationale for *UGT1A1* genotyping is explained. The authors reported that  *UGT1A1* plays a crucial role in the metabolism and elimination of SN-38. | **No**  The scientific background or rationale for *UGT1A1* genotyping is not explained. |
|  | 3 | Provide reasons for choosing the genes and SNPs genotyped. | **No**  Reasons for choosing the *UGT1A1* gene and the genotyped variants were not provided | **Yee**  Reasons for choosing the *UGT1A1* gene and the genotyped variant were provided | **Yes**  Reasons for choosing the *UGT1A1* gene and the **28* genotyped variant were provided. In particular, *UGT1A1*28* was genotyped because this polymorphism leads to reduced UGT1A1 activity in homozygous subjects, i.e. ~10% of North American population. In addition, this polymorphism increases the risk of neutropenia and diarrhea with irinotecan therapy. | **No**  Reasons for choosing the *UGT1A1* gene and the genotyped variants were not provided |
| **Objectives** | 4 | State specific objectives, including any prespecified hypotheses | **No** | **No** | **No** | **No** |
|  | 5 | State if the study is the first report of a pharmacogenetic association, a replication effort, or both. | **Yes**  Please refer to item 12 | **Yes**  Please refer to item 12 | **Yes**  Please refer to item 12 | **Yes**  Please refer to item 12 |
| **METHODS** |  |  |  |  |  |  |
| **Study Design** | 6 | Present key elements of study design early in the paper. | **Yes**  Key elements of the study design are reported as it follows: “*IMMU-132-01 was a single-arm, open-label, multicenter phase I/II trial comprising a dose-escalation and a cohort-expansion phase*”. | **Yes**  Key elements of the study design are reported as it follows: ‘*TROPHY-U-01 is an international, multicohort, open-label, phase II study of SG in patients with unresectable LA or* mUC’ | **Probably Yes**  Key elements of the study design are reported as it follows: ‘*Detailed methods for ASCENT (NCT02574455; registered October 12, 2015), a phase 3 randomized, open-label multicenter study, have been reported.*’ | **Probably Yes**  Key elements of the study design are reported as it follows: ‘*Details of the TROPiCS-02 trial, including access to the protocol, have been previously reported*. *Briefly, TROPiCS-02 was a global, randomised, open-label, multicenter, phase 3 study of sacituzumab govitecan versus treatment of physician’s choice in patients with locally recurrent inoperable or metastatic HR^+^ and HER2^–^ breast cancer*’. |
| **Setting** | 7 | Describe the setting, locations, and relevant dates, including periods of recruitment,  follow-up, and data collection | **Yes**  The paper briefly mentions that the study was conducted at multiple centers in the USA. Relevant dates, including periods of recruitment, follow-up, and data collection are reported in the Results’ section. However, detailed information on this trial can be found in the clinicaltrials.gov database. The study started on 17/12/2012 and was completed on 13/08/2020. The outcome measures were assessed up to the data cutoff date of 01/03/2019. | **Yes**  The data cut-off date for the updated analysis presented in the paper and the median follow-up are provided in the Results’ section. However, detailed information on this trial can be found in the clinicaltrials.gov database. This is a multicenter study carried out at 135 locations in the USA, Europe, the UK and South Korea. The study started on 13/08/2013. It is currently recruiting. Its completion is estimated by June 2030. | **Yes**  This information is not reported in this paper. However, detailed information on this trial can be found in the clinicaltrials.gov database. This is a multicenter study carried out at 230 locations in locations in North America (USA and Canda), Europe, and the UK. The study started on 07/11/2017 and was completed on 08/12/2020. The outcome measures were assessed up to the data cutoff date of 11/03/2020. | **Yes**  The authors report that ‘*TROPiCS-02 enrolled patients at 91 centres across North America (the USA and Canada) and Europe (Belgium, France, Germany, Italy, the Netherlands, Spain, and the UK)*’. Relevant dates, including periods of recruitment, follow-up, and data collection are reported in the Abstract and Results’ sections. Moreover, further information on this trial can be found in the dlinicaltrials.gov database.  This is a multicenter study carried out at 113 locations in North America (USA and Canada), Europe, and the UK. The study started on 08/05/2019 and was completed on 20/10/2023. The outcome measures were assessed up to the data cutoff date of 01/07/2022. |
| **Participants** | 8 | Give the eligibility criteria and the sources and methods of selection of participants. For a cohort study, describe methods of follow-up. For a case-control study, state whether true controls or population controls were used. Give the rationale for the choice of cases and controls. | **Probably Yes**  ***Main inclusion criteria reported in the paper:***  Individuals ≥ 18 years of age, with metastatic cancer (cervical, clear-cell renal, epithelial ovarian, endometrial, esophageal, gastric, hepatocellular, pancreatic ductal adenocarcinoma, squamous cell head and neck, thyroid, UC cancer; glioblastoma multiforme; mTNBC or non-mTNBC; and SCLC or NSCLC) who had relapsed after or were refractory to at least one prior standard therapeutic regimen. All subjects had measurable disease with a washout period of at least 2 weeks and ECOG performance status of 0 or 1.  ***Relevant exclusion criteria for the metanalysis:***  Gilbert’s disease, intolerance to previous irinotecan^1^  ***Primary outcome measures:*** SG safety and efficacy (ORR) (clinicaltrials.gov). SG pharmacokinetics was also studied.  ***Methods of follow-up***  Follow-up was performed through radiological imaging (CT or MRI). First scan was obtained at baseline (within 4 weeks of study entry) and assessments were performed every 8 weeks thereafter, with confirmatory CT/MRI scans 4 to 6 weeks after an initial partial response or complete response, until radiologic evidence of progression of disease. Exposure to SG was assessed in the OSP by evaluating treatment duration, number of doses, and number of treatment cycles. In addition, safety of SG in the OSP was evaluated based on AEs, laboratory assessments, physical examination, vital signs, and 12-lead electrocardiograms. AEs were classified according to the Medical Dictionary for Regulatory Activities version 20.0, with severity graded according to Common Terminology Criteria for Adverse Events version 4.3. | **Probably Yes**  ***Inclusion criteria for***  ***the Cohort 1 reported in the paper:***  Adults with  histologically documented LA or mUC (UC-predominant histology) who progressed after prior platinum-based (cisplatin or carboplatin) and CPI-based therapies in the first-line setting or within 12 months from the end of (neo) adjuvant chemotherapy for localized muscle-invasive UCwere eligible. Individuals with 0-1 ECOG, 3-month life expectancy, and adequate organ function.  ***Relevant exclusion criteria for the metanalysis:***  None  ***Primary outcome measures:*** Efficacy (ORR by independent review board).  ***Methods of follow-up:***  Follow-up was performed through CT or MRI evaluation. Scans were obtained at baseline and every 6 weeks from the initiation of treatment until the completion of 12 cycles; thereafter, scans could be obtained every 9 weeks. Confirmatory scans were obtained 4-6 weeks after initial evidence of response. | **Probably Yes**  ***Main inclusion criteria reported in the paper***:  Subjects with mTNBC (per American Society of Clinical Oncology/College of American Pathologists criteria) who relapsed after or were refractory to ≥2 previous standard chemotherapy regimens (no upper limit) for unresectable, locally advanced or metastatic disease. Patients with brain metastases stable for ≥4 weeks before treatment were eligible.  ***Relevant exclusion criteria for the metanalysis:***  Gilbert's disease.  ***Primary outcome measures:*** Efficacy (PFS by Independent Review Committee Assessment in Brain Metastasis Negative Population)  ***Methods of follow-up:***  Follow-up was performed through radiological imaging (CT or MRI), performed every 6 weeks for 36 weeks, then every 9 weeks thereafter, until disease progression leading to treatment discontinuation^15^. Responses required confirmatory scans 4 to 6 weeks later. Individuals were contacted every 4 weeks to assess survival during long-term follow-up. | **Probably Yes**  ***Main inclusion criteria reported in the paper***:  Subjects with confirmed HR^+^ and HER2^−^ locally recurrent inoperable or metastatic breast cancer who received at least one previous endocrine therapy, a taxane, and a CDK4/6 inhibitor in any setting and two to four previous chemotherapy regimens for metastatic disease. Adult individuals with 0-1 ECOG, at least one measurable target lesion.  ***Note***, bilirubin ≤ 3 institutional upper limits of normal for subjects with known Gilbert's syndrome.  ***Relevant exclusion criteria for the metanalysis:***  Previous treatment with topoisomerase 1 Inhibitors (free form or other formulations).  ***Primary outcome measures:***  Efficacy (PFS by Blinded Independent Central Review Assessment)  ***Follow-up*** was performed through radiological imaging (CT or MRI), performed every 6 weeks for the first 54 weeks and every 12 weeks thereafter^16^. |
|  | 9 | Report the drug and regime participants were exposed to and the length of exposure | **Yes**  Intravenous infusions of SG (8, 10, 12, or 18 mg/kg) on days 1 and 8 of 21-day cycles until disease progression or unacceptable toxicity, death, or withdrawal of consent | **Yes**  SG was administered at 10 mg/kg intravenously on days 1 and 8 of a 21-day cycle until progression, unacceptable toxicity, or withdrawal of informed consent. | **Yes**  Subjects were randomized 1:1 to SG 10 mg/kg intravenously on Days 1 and 8 of each 21-day cycle, or TPC (eribulin; vinorelbine; capecitabine; or gemcitabine) and received treatment until disease progression or unacceptable toxicity. Patients were stratified at randomization according to the number of previous chemotherapy regimens for advanced disease (2 or 3 vs. >3), the presence of known brain metastases at baseline (yes vs. no), and geographic region (North America vs. rest of the world). | Yes  Subjects were randomly assigned 1:1 to receive 10 mg/kg of SG intravenously on days 1 and 8 of each 21-day cycle or chemotherapy of physician’s choice determined before random assignment (eribulin, capecitabine, gemcitabine, or vinorelbine). Random assignment was stratified by number of prior chemotherapy regimens for metastatic disease, visceral metastases, and prior endocrine treatment in the metastatic setting for at least 6 months. Patients were treated until disease progression, unacceptable toxicity, withdrawal of consent, or per investigator’s decision. Treatment beyond progression was permitted if considered clinically useful by the investigator. |
|  | 10 | For a matched case-control study, give matching criteria and the number of controls  per case. | **Not applicable** | **Not applicable** | **Not applicable** | **Not applicable** |
|  | 11 | Give information on the criteria and methods for selection of subsets of participants  from a larger study, when relevant. | **Not applicable**  *UGT1A1* testing was required for all subjects enrolled in the study, although the association between *UGT1A1* and safety was not the main clinical outcome, nor subjects were enrolled and population stratified based on the *UGT1A1* status. | **Not applicable**  *UGT1A1* testing was required for all subjects enrolled in the study, although the association between *UGT1A1* and safety was not the main clinical outcome, nor subjects were enrolled and population stratified based on the *UGT1A1* status. | **Not applicable**  *UGT1A1* testing was required for all subjects enrolled in the study, although the association between *UGT1A1* and safety was not the main clinical outcome, nor subjects were enrolled and population stratified based on the *UGT1A1* status. | **Not applicable**  *UGT1A1* testing was required for all subjects enrolled in the study, although the association between *UGT1A1* and safety was not the main clinical outcome, nor subjects were enrolled and population stratified based on the *UGT1A1* status. |
|  | 12 | If other publications report results for the same patient cohort or a subset of the  patient cohort, provide information on this patient cohort overlap and references to  the relevant publications. | **Probably Yes**  Other three publications reported data from this trial regarding the association between *UGT1A1* and SG toxicity: 1) Ocean AJ et al., 2017^1^: *UGT1A1* genotype was available for 146; 2) Kalinsky K et al., 2020^2^: *UGT1A1* genotype was available for 48; 3) Bardia A et al., 2019^3^: *UGT1A1* genotype was available for 333. The study references both previous papers but the overlap with these two sub-cohorts is not clear | **Yes**  A previous publication reported data from this trial regarding the association between *UGT1A1* and SG toxicity^17^. The total number of *UGT1A1* genotyped individuals was 105 vs 106 in this study^12^. The study references the previous one and the overlap is almost complete. | **No** | **No** |
|  | 13 | Report disease/clinical indication of patients using a standardized ontology when  possible. | **Yes**  Relapsed or refractory metastatic epithelial cancer, including TNBC (n=144), BC (n=68), small cell lung cancer (n=62), non-small cell lung cancer (n=54), UC (n=49), colorectal (n=31), oesophageal (n=19), endometrial (n=18) cancers, pancreatic ductal adenocarcinoma (n=16), castrate-resistant prostate carcinoma (n=11), epithelial ovarian carcinoma (n=8), gastric adenocarcinoma (n=5), glioblastoma multiforme (n=3), squamous cell head and neck (n=3) hepatocellular (n=2), cervical (n=1), clear-cell renal (n=1) cancers | **Yes**  Relapsed locally advanced or metastatic UC | **Yes**  Relapsed or refractory metastatic TNBC, according to standard American Society of Clinical Oncology - College of American Pathologists criteria | **Yes**  Relapsed or refractory confirmed HR^+^/HER2^−^ locally recurrent inoperable or metastatic breast cancer |
| **Variables** | 14 | Clearly define all outcomes, potential confounders, and effect modifiers. Give  diagnostic criteria, if applicable | **Probably Yes**  The evaluation of the pharmacogenetic association between *UGT1A1* and safety was not the primary outcome of the study, however some relevant information can be extracted from the text.  ***Potential confounders***: low absolute neutrophil count and hemoglobin at baseline due to previous therapies; impaired renal and liver function; previous toxicity to irinotecan.  ***Potential effect modifiers***: previous chemotherapies  ***Diagnostic criteria for the adverse events*** were based on the Medical Dictionary for Regulatory Activities version 20.0, with severity graded according to Common Terminology Criteria for Adverse Events version 4.3. | **Probably Yes**  The evaluation of the pharmacogenetic association between *UGT1A1* and safety was not the primary outcome of the study, however some relevant information can be extracted from the text.  ***Potential confounders***: low absolute neutrophil count and hemoglobin at baseline due to previous therapies; impaired renal and liver function; previous toxicity to irinotecan.  ***Potential effect modifiers***: previous chemotherapies  ***Diagnostic criteria for the***  ***adverse events*** were based Medical Directory for Regulatory Activities (MedDRA) Version 22 or greater, with severity graded according to the National Cancer Institute Common Terminology Criteria for Adverse Events (NCI-CTCAE) v5.0. | **Probably Yes**  The evaluation of the pharmacogenetic association between *UGT1A1* and safety was not the primary outcome of the study, however some relevant information can be extracted from the text  ***Potential confounders***: low absolute neutrophil count and hemoglobin at baseline due to previous therapies; impaired renal and liver function; previous toxicity to irinotecan.  ***Potential effect modifiers***: previous chemotherapies  ***Diagnostic criteria for the adverse events*** were coded per the Medical Dictionary for Regulatory Activities, version 22.1. and assessed per the National Cancer Institute Common Terminology Criteria for AEs, version 4.03. | **Probably Yes**  The evaluation of the pharmacogenetic association between *UGT1A1* and safety was not the primary outcome of the study, however some relevant information can be extracted from the text  ***Potential confounders***: low absolute neutrophil count and hemoglobin at baseline due to previous therapies; impaired renal and liver function; previous toxicity to irinotecan.  ***Potential effect modifiers***: previous chemotherapies  ***Severity of adverse events*** was graded using National Cancer Institute Common Terminology Criteria for Adverse Events v5.0 |
|  | 15 | Provide justification for choice of outcomes. | **Probably Yes**  The assessment of the pharmacogenetic association was an exploratory outcome. | **Probably Yes**  The assessment of the pharmacogenetic association was an exploratory outcome. | **Probably Yes**  The assessment of the pharmacogenetic association was an exploratory outcome. | **Probably Yes**  The assessment of the pharmacogenetic association was an exploratory outcome. |
|  | 16 | Clearly define genetic exposures (genetic variants) using a widely used nomenclature  system. | **Yes**  In the supplementary material, it is reported that ‘*the UGT1A1*28 allele is associated with decreased enzyme activity. Prolonged SN-38 exposure has been observed in UGT1A1*28 homozygous subjects treated with irinotecan. These subjects are at higher risk of severe neutropenia and diarrhoea when receiving irinotecan or its metabolites*’. | **Yes** | **Yes**  In the Introduction, it is reported that ‘*the enzyme UGT1A1 plays a key role in detoxifying SN-38 by glucuronidation, producing a metabolite that is then eliminated from the body primarily by biliary excretion. UGT1A1 activity is reduced in the UGT1A1 *28/*28 phenotype, which is found in approximately 10% of North American patients; this polymorphism leads to a higher risk of neutropenia and diarrhea with irinotecan therapy*’. | **Yes**  In the Discussion, it is reported that ‘*polymorphisms in UGT1A1 have been associated with increased incidence of known adverse events, such as neutropenia, febrile neutropenia, anemia, and diarrhoea, following treatment with some systemic anticancer agents, including sacituzumab govitecan, because of the reduced rate of SN-38 glucuronidation’*. |
|  | 17 | Report the rs number of each genotyped SNP | **No**  The main UGT1A1 variant analyzed is rs8175347(TA)7 (UGT1A1*28) also known as rs3064744. This information was not reported in the study. | **Yes**  The rs8175347 associated with the UGT1A1*28 variant is reported in the Methods | **No**  This information was not reported in the study. | **No**  This information was not reported in the study. |
|  | 18 | Clearly state how haplotypes or star alleles were defined. | **No**  The rs8175347(TA)7 is a promoter variant which affects the TATA box region of the *UGTA1A* gene. It is characterized by the insertion of an extra TA repeat (c.-39_-40 ins TA) in the TATA box thus reducing gene expression^18^. This information is not reported in the study. | **No**  This information is not reported in the study. | **No**  This information is not reported in the study. | **No**  This information is not reported in the study. |
|  | 19 | If referring to the minor, major, wild-type, mutant, reference, risk or effect allele of a  variant, state which allele this is and for which given population/cohort. | **Probably Yes**  The wild type allele is *UGT1A1***1*, that is characterized by 5 TA repeats in the TATA box region. The *UGT1A1***28* is the minor allele variant (MAF=0.29-0.45 in Caucasians, MAF=0.42-0.51 in Africans, MAF=0.09-0.16 in Asians)^18^. Only a broad definition of wild-type and mutant type is provided in the study. | **Probably Yes**  Only a broad definition of wild-type and mutant type is provided in the study. | **Probably Yes**  Only a broad definition of wild-type and mutant type is provided in the study. | **Probably Yes**  Only a broad definition of wild-type and mutant type is provided in the study. |
| **Data sources/ measurement** | 20 | For each variable of interest, give sources of data and details of methods of assessment (measurement). Describe comparability of assessment methods if there is  more than one group. | **Probably Yes**  Blood samples were collected for *UGT1A1* genotyping prior to treatment. Genotype analysis was performed initially by allele-specific amplification of the *UGT1A1* promoter region followed by gel electrophoresis and documentation of the amplification product with the *UGT1A1* Genotyping Kit from EntroGen, Inc. (Woodland Hills, CA) and after mid-2015 by Covance Laboratories (Redmond, WA) via polymerase chain reaction–based assay and Sanger sequencing with Covance VAR0106 (Bardia A et al., 2019). | **Probably Yes**  A blood sample was provided to assess *UGT1A1* status. *UGT1A1* rs8175347 (**28*) was assessed by Sanger sequencing assay. | **Probably Yes**  At baseline, a single whole blood sample was collected from all subjects receiving SG for UGT1A1 genotyping for retrospective assessment of safety.  *UGT1A1* genotype was determined by Sanger sequencing, performed  Centrally. | **Probably Yes**  At baseline, a single whole-blood sample was collected from all subjects receiving SG for *UGT1A1* genotyping for retrospective assessment of safety.  UGT1A1 genotype was determined by Sanger sequencing (platform ABI 3500XL Genetic Analyzer; Applied Biosystems, Waltham, MA, USA; software ABI Data Collection Software; ABI Sequence Analysis Software, Waltham, MA, USA) |
|  | 21 | Describe laboratory methods, including source and storage of DNA, genotyping methods and platforms (including the allele calling algorithm used, and its version), error rates, and call rates. State the laboratory/center where genotyping was done. Describe comparability of laboratory methods if there is more than one group. Specify whether genotypes were assigned using all of the data from the study simultaneously or in smaller batches. | **No**  This information was not provided | **No**  This information was not provided | **No**  This information was not provided | **Probably No**  Only mentioned that genotyping was performed centrally at Covance Genomics Lab (LabCorp, Burlington, NC, USA). |
|  | 22 | Describe genotype quality control methods and findings. | **No**  This information was not provided | **No**  This information was not provided | **No**  This information was not provided | **No**  This information was not provided |
|  | 23 | For quantitative outcome variables, specify if any investigation of potential bias resulting from pharmacotherapy was undertaken. If relevant, describe the nature and magnitude of the potential bias, and explain what approach was used to deal with this. | **No**  This information was not provided. However, no quantitative variables were included in the present meta-analysis | **No**  This information was not provided. However, no quantitative variables were included in the present meta-analysis | **No**  This information was not provided. However, no quantitative variables were included in the present meta-analysis | **No**  This information was not provided. However, no quantitative variables were included in the present meta-analysis |
|  | 24 | Report how adherence to treatment was assessed, and report the results of the assessment. | **Probably Yes**  Detailed information was not provided. However, the OSP included all subjects who received at least one dose of SG, regardless of cancer type, number of previous anticancer treatments, or SG dose received | **Probably Yes**  Detailed information was not provided. However, all patients who received ≥1 dose of SG were included in the evaluation of safety | **Probably Yes**  Detailed information was not provided. However, the OSP consisted of subjects (including those with brain metastases) who received at least one dose of study treatment, either SG or TPC | **Probably Yes**  Detailed information was not provided. However, The OSP consisted of subjects who received at least one dose of study treatment, either SG or TPC |
| **Study size** | 25 | Explain how the study size was arrived at, or provide details of the a priori power to detect effect sizes of varying degrees. | **No**  An *a priori* statistical analysis was not performed to establish sample size and power for detecting the pharmacogenetic association between *UGT1A1**28 and different SG toxicities.  ***Note:*** *UGT1A1* genotype analysis was conducted as an exploratory objective^3^. | **No**  An *a priori* statistical analysis was not performed to establish sample size and power for detecting the pharmacogenetic association between *UGT1A1**28 and different SG toxicities. | **No**  An *a priori* statistical analysis was not performed to establish sample size and power for detecting the pharmacogenetic association between *UGT1A1**28 and different SG toxicities.  ***Note:*** Exploratory endpoints included safety by UGT1A1 variant status^13^. | **No**  An *a priori* statistical analysis was not performed to establish sample size and power for detecting the pharmacogenetic association between *UGT1A1**28 and different SG toxicities. |
|  | 26 | Explain how quantitative variables (confounders and effect modifiers) were handled in the analyses. If applicable, describe which groupings were chosen, and why. | **Not Applicable**  Only the number and the percentage in each UGT1A1 genetic subgroup of adverse events per genotype was provided. Thus, raw counts were extracted rather than adjusted analyses. | **Not Applicable**  Only the number (or the percentage in each UGT1A1 genetic subgroup) of adverse events per genotype was provided. Thus, raw counts were extracted rather than adjusted analyses. | **Not Applicable**  Only the number (or the percentage in each UGT1A1 genetic subgroup) of adverse events per genotype was provided. Thus, raw counts were extracted rather than adjusted analyses. | **Not Applicable**  Only the number (or the percentage in each UGT1A1 genetic subgroup) of adverse events per genotype was provided. Thus, raw counts were extracted rather than adjusted analyses. |
| **Statistical methods** | 27  a) | ***Address the followings***:  Describe methods used to control for confounding | **Probably No**  Control of potential confounders was achieved by excluding from the study subjects: with inadequate hematologic function, particularly absolute neutrophil count ≤1500/mm^3^ and hemoglobin ≤9 g/dL; reduced renal function, creatinine >2.0x upper limit of normal (ULN); and reduced hepatic function, bilirubin >1.5x ULN, aspartate aminotransferase and alanine aminotransferase >3x ULN or 5x ULN in patients with known liver metastases. Other relevant exclusion criteria for the control of confounding were a known history of anaphylactic reaction to irinotecan or grade ≥3 gastrointestinal toxicity to prior irinotecan | **Probably No**  Control of potential confounders was achieved by including in the study subjects with adequate renal and hepatic function; and adequate hematologic parameters without transfusional support. | **Probably No**  Control of potential confounders was achieved by including in the study subjects with adequate renal and hepatic function; and adequate hematologic parameters without transfusional support.  In addition, inhibitors and inducers of *UGT1A1* were used with caution. | **Probably No**  Control of potential confounders was achieved by including in the study subjects with adequate renal and hepatic function, and adequate hematologic parameters.  In addition, subjects that were previously treated with a topoisomerase 1 inhibitor therapy were excluded from the trial. |
|  | b) | Describe any methods used to examine subgroups and interactions. | These methods were not reported | These methods were not reported | These methods were not reported | These methods were not reported |
|  | c) | Explain how missing data were addressed. | Genotype was not done or missing for 92/495 enrolled subjects, although no further details were provided. | Genotype was missing for 8/113 enrolled subjects. | Genotype was reported as unknown/other in 15/258 enrolled subjects in the SG arm. | Genotype was reported for all subjects (268) included in the safety population. |
|  | d) | Cohort study - If applicable, explain how loss to follow-up was addressed | The Overall Safety Population included all patients who received at least one dose of SG. | The Overall Safety Population included all patients who received at least one dose of SG. | The safety population consisted of 482 subjects (of whom 53 had brain metastases) who received at least one dose of study treatment, including 258 in the SG arm and 224 in the TPC arm. | The safety population consisted of 517 subjects who received at least one dose of study treatment, including 268 in the SG arm and 249 in the TPC arm. |
|  | e) | Case-control study—If applicable, explain how matching of cases and controls was  addressed. | **Not Applicable** | **Not Applicable** | **Not Applicable** | **Not Applicable** |
|  | f) | Describe any sensitivity analyses. | Sensitivity analyses related to the pharmacogenetic association were not performed | Sensitivity analyses related to the pharmacogenetic association were not performed | Sensitivity analyses related to the pharmacogenetic association were not performed | Sensitivity analyses related to the pharmacogenetic association were not performed |
|  | 28 | State whether Hardy–Weinberg equilibrium was considered, and if so, how. | **No**  This aspect was not considered. The *UGT1A1*28/*28* genotype was found in 9.3% of the genotyped individuals. The reported frequency in Caucasians is 8-20%^18^. | **No**  This aspect was not considered. The *UGT1A1*28/*28* genotype was found in 12% of the genotyped individuals. | **No**  This aspect was not considered. The *UGT1A1*28/*28* genotype was found in 13% of the genotyped individuals. | **No**  This aspect was not considered. The *UGT1A1*28/*28* genotype was found in 9% of the genotyped individuals. |
|  | 29 | Describe any methods used for inferring genotypes or haplotypes | **No**  Not Described for the pharmacogenetic analysis. | **No**  Not Described for the pharmacogenetic analysis. | **No**  Not Described for the pharmacogenetic analysis. | **No**  Not Described for the pharmacogenetic analysis. |
|  | 30 | Describe any methods used to assess or address population stratification | **No**  Not Described for the pharmacogenetic analysis.  The study enrolled mainly White individuals (81.8%). | **No**  Not Described for the pharmacogenetic analysis.  The study enrolled mainly White individuals (74%). | **No**  Not Described for the pharmacogenetic analysis.  The study enrolled mainly White individuals (80%). | **No**  Not Described for the pharmacogenetic analysis.  The study enrolled mainly White individuals (67%). |
|  | 31 | Describe any methods used to assess and correct for relatedness among subjects.  Report results of assessments for relatedness. | **No**  Not Described. Individuals were most likely not related | **No**  Not Described. Individuals were most likely not related | **No**  Not Described. Individuals were most likely not related | **No**  Not Described. Individuals were most likely not related |
|  | 32 | Describe any methods used to address multiple comparisons or to control risk of  false positive results due to (a) multiple genetic variants, (b) multiple outcomes, and  (c) multiple assumptions regarding mode of inheritance. | **No**  Not Described | **No**  Not Described | **No**  Not Described | **No**  Not Described |
|  | 33 | Describe any methods used to adjust for extent of adherence in the analyses | **Probably Yes**  Not Described. However, the OSP included all patients who received at least one dose of SG. | **Probably Yes**  Not Described. However, the OSP included all patients who received at least one dose of SG. | **Probably Yes**  Not Described. However, the OSP consisted of 482 patients (of whom 53 had brain metastases) who received at least one dose of study treatment, including 258 in the SG arm and 224 in the TPC arm. | **Probably Yes**  Not Described. However, the OSP consisted of 517 patients who received at least one dose of study treatment, including 268 in the SG arm and 249 in the TPC arm. |
| **RESULTS** |  |  |  |  |  |  |
| **Participants** | 34 | Report the numbers of individuals at each stage of the study — e.g., numbers potentially eligible, examined for eligibility, confirmed eligible, included in the study, completing follow-up, and analyzed | **Probably No**  The paper provides only number of subjects genotyped for *UGT1A1* (403/495 overall safety population). | **Probably No**  The papers provide only the number of subjects genotyped for *UGT1A1* (105/113 overall safety population^17^; 106/113 overall safety population^12^). | **Probably No**  The paper provides the number of subjects genotyped for *UGT1A1* in the SG treatment arm (243/258 overall safety population). | **Probably No**  The paper provides the number of subjects genotyped for *UGT1A1* in the SG treatment arm (100% of the overall safety population). |
| **SNPs** | 35 | Report any SNPs that were excluded from analysis, and provide reasons for these exclusions. | **Not Applicable**  The focus was on variants defining *UGT1A1*28*.  Genotyping was not done or missing in 92 (18.6%) individuals out of 495 subjects included in the overall safety population. No reasons were provided. | **Not Applicable**  The focus was on variants defining *UGT1A1*28*.  Genotyping was missing for 7 (6%) individuals out of 113 subjects included in the overall safety population^12^. No reasons were provided. | **Not Applicable**  The focus was on variants defining *UGT1A1*28*.  Genotyping was uknown or other in 15 (6%) individuals out of 258 subjects included in the OSP. No reasons were provided. | **Not Applicable**  The focus was on variants defining *UGT1A1*28*.  However, 3 individuals (1%) treated with SG carried other genotypes, one each of **1/*36*, **1/*37*, and **28/*36*..  ***Note***: **36* is considered functionally equal to **1*; **37* is considered functionally equal to **28* |
| **Descriptive data** | 36 | Give characteristics of study participants (e.g., demographic, clinical, social, ethnicity) and information on potential confounders. | **No**  Demographics and other relevant information were provided only for the OSP | **No**  Demographics and other relevant information were provided only for the OSP | **No**  Demographics and other relevant information were provided only for the OSP | **No**  Demographics and other relevant information were provided only for the OSP |
|  | 37 | Cohort study—Summarize follow-up time, e.g., average and/or total amount | **Yes**  At the data cutoff for the final analysis (1/03/2019), the median follow-up was 8.97 months (range: 0.26-55.72). | **Yes**  At the data cutoff for this interim analysis (26/07/2022), the median follow-up was 10.5 months (range: 0.3-40.9). | **No**  The median follow up was not reported in the paper^13^. In the SG arm, median relative dose intensity was 99.7%. | **Yes**  The median follow up was 13.8 months (range: 8.3 -19.8) for the SG group. |
|  | 38 | Where HWE tests have been undertaken, highlight SNPs that deviate from HWE. | **No**  No specific tests were undertaken to assess deviation from HWE (please refer to criterion 28) | **No**  No specific tests were undertaken to assess deviation from HWE (please refer to criterion 28) | **No**  No specific tests were undertaken to assess deviation from HWE (please refer to criterion 28) | **No**  No specific tests were undertaken to assess deviation from HWE (please refer to criterion 28) |
|  | 39 | Where population stratification is assessed, report the results. | **No**  Not assessed (please refer to criteria 30-31) | **No**  Not assessed (please refer to criteria 30-31) | **No**  Not assessed (please refer to criteria 30-31) | **No**  Not assessed (please refer to criteria 30-31) |
| **Outcome data** | 40a | For a cohort study, report all outcomes (phenotypes) investigated for each genotype category over time. | **Probably Yes**  These data were available for most of the primary outcomes included in the meta-analysis for all *UGT1A1* genotyped subjects enrolled in the studies at the data cutoff date or during interim analyses | **Probably Yes**  These data were available for most of the primary outcomes included in the meta-analysis for all *UGT1A1* genotyped subjects enrolled in the studies at the data cutoff date or during interim analyses | **Probably Yes**  These data were available for most of the primary outcomes included in the meta-analysis for all *UGT1A1* genotyped subjects enrolled in the studies at the data cutoff date or during interim analyses | **Probably Yes**  These data were available for most of the primary outcomes included in the meta-analysis for all *UGT1A1* genotyped subjects enrolled in the studies at the data cutoff date or during interim analyses |
|  | 40b | For a case-control study, report numbers in each genotype category for all outcomes investigated. | Not applicable | Not applicable | Not applicable | Not applicable |
|  | 40c | For a cross-sectional study, report all outcomes (phenotypes) investigated for each genotype category | Not applicable | Not applicable | Not applicable | Not applicable |
|  | 41 | If a study includes more than one ethnic group, provide the summary data specified in (40) per ethnic group. | **No**  The study included more than 1 ethnic group. However, pharmacogenetic data were not corrected for the specific ethnicity. | **No**  The study included more than 1 ethnic group. However, pharmacogenetic data were not corrected for the specific ethnicity. | **No**  The study included more than 1 ethnic group. However, pharmacogenetic data were not corrected for the specific ethnicity. | **No**  The study included more than 1 ethnic group. However, pharmacogenetic data were not corrected for the specific ethnicity. |
| **Main results** | 42 | Give unadjusted estimates, and if applicable, confounder-adjusted estimates and their precision (e.g., 95% confidence intervals). Make clear which confounders were adjusted for and why they were included. | **Probably yes**  Unadjusted estimates were provided | **Probably yes**  Unadjusted estimates were provided | **Probably yes**  Unadjusted estimates were provided | **Probably yes**  Unadjusted estimates were provided |
|  | 43 | Report category boundaries when continuous variables were categorized. | **Not Applicable** | **Not Applicable** | **Not Applicable** | **Not Applicable** |
| **Other Analyses** | 44 | Report other analyses done—e.g., analyses of subgroups and interactions, and sensitivity analyses. | **Not Applicable** | **Not Applicable** | **Not Applicable** | **Not Applicable** |
|  | 45 | If numerous genetic exposures (genetic variants) were examined, summarize results from all analyses undertaken. | **Not Applicable** | **Not Applicable** | **Not Applicable** | **Not Applicable** |
|  | 46 | If detailed results are available elsewhere, i.e., in supplementary materials, state how they can be accessed. | **Yes**  Data used in the meta- analysis were extracted using Figure 1 and Supplementary Table 3^11^. | **Yes**  Data used in the meta- analysis were extracted using Figure 3^12^. Data on severe (≥ grade 3) neutropenia were found in the Results, Safety^17^. | **Yes**  Data used in the meta- analysis were extracted using Table 4^13^. | **Yes**  Data used in the meta- analysis were extracted using Supplementary Table 8^14^. |
| **DISCUSSION** |  |  |  |  |  |  |
| **Key results** | 47 | Summarize key results with reference to study objectives. | **Probably Yes**  The pharmacogenetic association between the *UGT1A1* status and SG toxicity was not a primary outcome of the study. Nevertheless, a paragraph discussing the *UGT1A1* data was included in the Discussion section^11^. | **Probably Yes**  The pharmacogenetic association between the *UGT1A1* status and SG toxicity was not a primary outcome of the study. Nevertheless, a paragraph discussing the *UGT1A1* data was included in the Discussion section^12,17^. | **Probably Yes**  The pharmacogenetic association between the *UGT1A1* status and SG toxicity was not a primary outcome of the study. Genotyping was performed to all subjects enrolled in the SG treatment arm. Results were reported and discussed^13^. | **Probably Yes**  The pharmacogenetic association between the *UGT1A1* status and SG toxicity was not a primary outcome of the study. Genotyping was performed to all subjects enrolled in the SG treatment arm. Results were reported and discussed^14^. |
| **Limitations** | 48 | Discuss limitations of the study, taking into account sources of potential bias or imprecision. Discuss both direction and magnitude of any potential bias. | **No**  Not Discussed  Lack of pharmacokinetics data in the UGT1A1 genetic subgroups to assess the correlation between plasma SN-38 concentrations and toxicity. | **No**  Not Discussed  Low number of subjects with *UGT1A1*28* homozygous genotype (14/106, 12%)  Lack of randomization | **No**  Not Discussed  Low frequency of the *UGT1A1*28* homozygous genotype (34/243,13%). | **No**  Not Discussed  Low frequency of the *UGT1A1*28* homozygous (25/268, 9%). |
| **Interpretation** | 49 | Give a cautious overall interpretation of results considering objectives, limitations, multiplicity of analyses, results from similar studies, and other relevant evidence. | **Probably Yes**  Pharmacogenetic data from this trial were exploratory. However, these results suggest an increased risk of neutropenia in *UGT1A1*28* homozygous individuals. The paper advises close monitoring of subjects with known *UGT1A1*28* homozygous genotype treated with SG^11^. | **Probably Yes**  Pharmacogenetic data from this trial were exploratory. However, numerically higher dose interruptions were observed in *UGT1A1*28* homozygous subjects^12^. | **Probably Yes**  Pharmacogenetic data from this trial were exploratory. However, treatment discontinuation due to adverse effects  was more common in *UGT1A1*28* homozygous subjects in comparison to other genotypes  (6%, 1%, and 2%, respectively). | **Probably No**  Pharmacogenetic data from this trial were exploratory. |
| **Generalizability** | 50 | Discuss the generalizability (external validity) of the study results. | **Not Applicable**  Pretreatment *UGT1A1* genotyping was not clearly recommended based on the data of this trial^11^. | **Not Applicable**  Pretreatment *UGT1A1* genotyping is not recommended | **Not Applicable**  Despite the increased rate of discontinuation in *UGT1A1*28* homozygous individuals, the safety profile of SG was deemed favorable when using adequate toxicity management strategies, including active monitoring and early intervention with dose reductions and supportive medication use. The authors concluded that results from the trial confirmed the recommended SG starting dosage (10 mg/kg) in all *UGT1A1* genotype groups. | **Not Applicable** |
| **OTHER INFORMATION** |  |  |  |  |  |  |
| **Study registration** | 51 | State whether the study has been registered. If the study has been registered, provide details of the registry. | **Yes**  The study is registered in clinicaltrials.gov database as NCT01631552 | **Yes**  The study is registered in clinicaltrials.gov database as NCT03547973 | **Yes**  The study is registered in clinicaltrials.gov database as NCT02574455 | **Yes**  The study is registered in clinicaltrials.gov database as NCT03901339 |
| **Ethical Approval** | 52 | Report whether ethical approval was obtained for the collection of genetic data | **Yes**  Ethical approval was obtained by each institutional review board of the participating centres | **Yes**  The Protocol (online only) was approved by the institutional review boards or independent ethics committees at the participating institution | **Yes**  The study was approved by each institutional review board or ethics committee prior to initiation. | **Yes**  The study was approved by national regulatory authorities and each investigational site’s institutional review or ethics committee before implementation |
| **Funding** | 53 | Give the source of funding and the role of the funders for the present study, and if applicable, for the original study on which the present article is based. | **Yes**  ***Sponsor***: Gilead Sciences | **Yes**  ***Sponsor***: Gilead Sciences | **Yes**  ***Sponsor***: Gilead Sciences | **Yes**  ***Sponsor***: Gilead Sciences |
| **Database** | 54 | State whether databases for the analyzed data are or will become publicly available, and if so, how they can be accessed. | **Yes**  Data on UGT1A1 is available through international literature. | **Yes**  Data on UGT1A1 is available through international literature. | **Yes**  Data on UGT1A1 is available through international literature. | **Yes**  Data on UGT1A1 is available through international literature. |

***Abbreviations***: AE, Adverse event; BC, Hormone receptor positive and human epidermal growth factor receptor 2 positive/negative breast cancer; CPI, checkpoint inhibitor, CT, computed tomography; ECOG, Eastern Cooperative Oncology Group; HR, hormone receptor; HER2, human epidermal growth factor receptor 2; HWE, Hardy–Weinberg equilibrium; LA, locally advanced; MAF, minor allelic frequency; MRI, magnetic resonance imaging; mTNBC, metastatic triple negative breast cancer; mUC, metastatic urothelial carcinoma; ORR, objective response rate; OSP, overall safety population; rs, reference SNP cluster ID; SG, Sacituzumab Govitecan; SNP, single nucleotide polymorphism; TPC, treatment of physician's choice; UC, urothelial cancer or carcinoma; UDP, uridine diphosphate; UGT, UDP-glucuronosyltransferase.

**Supplementary Figures**

**
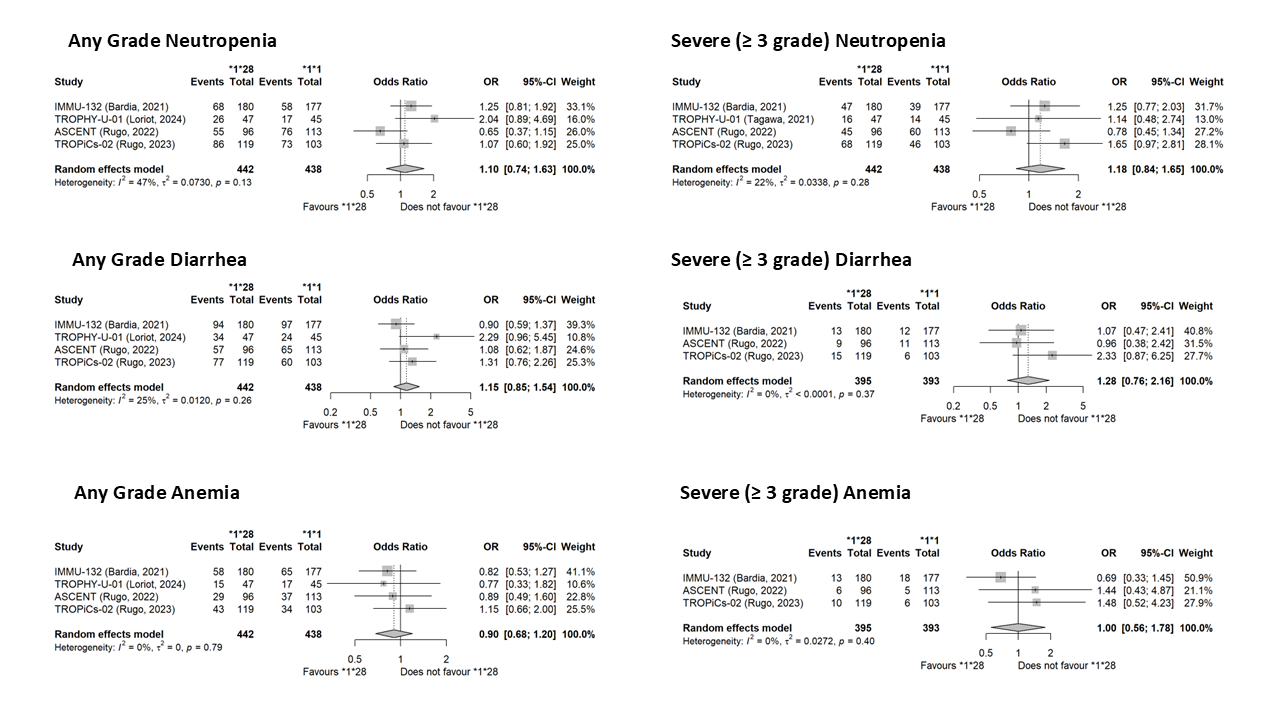
Figure S1.** Forest plots reporting the impact of the *UGT1A1*1/*28* genotype on the risk of developing different toxicities in comparison to wild-type subjects. The effect size is calculated as Odd Ratio (OR) and corresponding 95% confidence interval (95% CI). Data suggest no significant differences between the two genetic groups.

**Figure S2.** Forest plots reporting the impact of the *UGT1A1*28* variant on the risk of developing severe (≥ 3 grade) febrile neutropenia in response to SGs. The effect size is calculated as Odd Ratio (OR) and corresponding 95% confidence interval (95% CI). Data suggest a not-significant trend towards increased risk of developing severe (≥ 3 grade) febrile neutropenia toxicity in carriers of the *UGT1A1*28* variant, particularly in homozygous subjects.

**
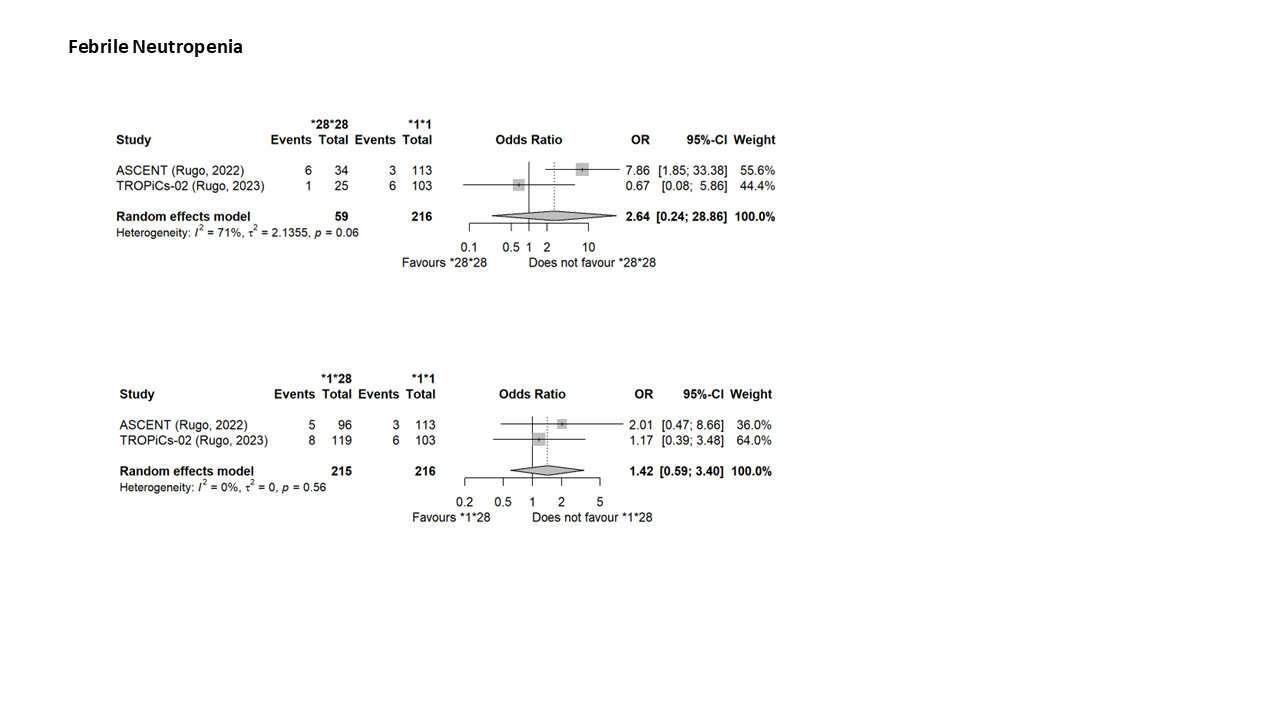
**

**Figure S3.** Forest plots reporting the impact of the *UGT1A1*28* heterozygosity on the likelihood of dose modification/suspension/interruption in subjects treated with SGs. The effect size is calculated as Odd Ratio (OR) and corresponding 95% confidence interval (95% CI).

**
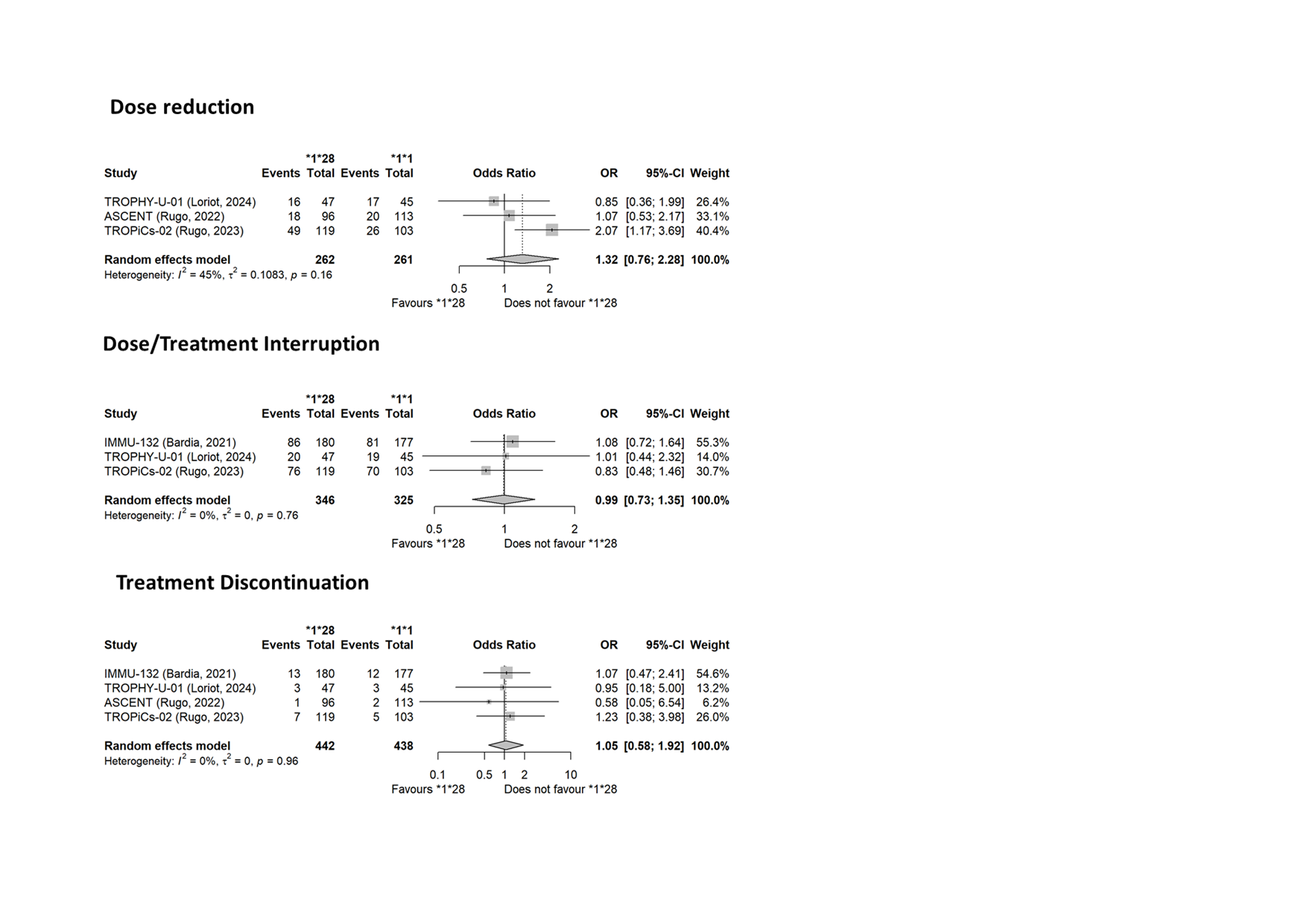
**

**Supplementary References**

1. Ocean, A.J., et al. Sacituzumab govitecan (IMMU-132), an anti-Trop-2-SN-38 antibody-drug conjugate for the treatment of diverse epithelial cancers: Safety and pharmacokinetics. *Cancer*, **123**: 3843-3854 (2017).
2. Kalinsky, K., et al. Sacituzumab govitecan in previously treated hormone receptor-positive/HER2-negative metastatic breast cancer: final results from a phase I/II, single-arm, basket trial. *Ann. Oncol*., **31**: 1709-1718 (2020).
3. Bardia, A., et al. Sacituzumab Govitecan-hziy in Refractory Metastatic Triple-Negative Breast Cancer. *N. Engl. J. Med*., **380**: 741-751 (2019).
4. Xu, B., et al. A Phase IIb, single arm, multicenter trial of sacituzumab govitecan in Chinese patients with metastatic triple-negative breast cancer who received at least two prior treatments. *Int. J. Cancer*, **152**: 2134-2144 (2023).
5. Naito, Y., et al. Preliminary results from ASCENT-J02: a phase 1/2 study of sacituzumab govitecan in Japanese patients with advanced solid tumors. *Int. J. Clin. Oncol*., **29**: 1684-1695 (2024).
6. Wong, M.H., et al. UGT1A1*28 polymorphism and the risk of toxicity and disease progression in patients with breast cancer receiving sacituzumab govitecan. *Cancer Med*., **13**: e70096 (2024).
7. Caputo, R., et al. Sacituzumab Govitecan for the treatment of advanced triple negative breast cancer patients: a multi-center real-world analysis. *Front. Oncol*., **14**: 1362641 (2024).
8. Page, M.J., et al. The PRISMA 2020 statement: an updated guideline for reporting systematic reviews. *B.M.J.*, **372**: n71 (2021).
9. Guyatt, G.H., et al. GRADE: an emerging consensus on rating quality of evidence and strength of recommendations. *B.M.J*.; **336**: 924-6 (2008)
10. Chaplin, M., et al. STrengthening the Reporting Of Pharmacogenetic Studies: Development of the STROPS guideline. *PLoS Med*., **17**: e1003344 (2020).
11. Bardia, A., et al. Sacituzumab govitecan, a Trop-2-directed antibody-drug conjugate, for patients with epithelial cancer: final safety and efficacy results from the phase I/II IMMU-132-01 basket trial. *Ann. Oncol.*, **32**: 746-756 (2021).
12. Loriot, Y., et al. TROPHY-U-01, a phase II open-label study of sacituzumab govitecan in patients with metastatic urothelial carcinoma progressing after platinum-based chemotherapy and checkpoint inhibitors: updated safety and efficacy outcomes. *Ann. Oncol.*, **35**: 392-401 (2024).
13. Rugo, H.S., et al. Safety analyses from the phase 3 ASCENT trial of sacituzumab govitecan in metastatic triple-negative breast cancer. *NPJ* *Breast Cancer*, **8**: 98 (2022).
14. Rugo, H.S., et al. Overall survival with sacituzumab govitecan in hormone receptor-positive and human epidermal growth factor receptor 2-negative metastatic breast cancer (TROPiCS-02): a randomised, open-label, multicentre, phase 3 trial. *Lancet*, **402**: 1423-1433 (2023).
15. Bardia, A., et al. Sacituzumab Govitecan in Metastatic Triple-Negative Breast Cancer. *N Engl J Med.*, **384**: 1529-1541 (2021).
16. Rugo, H.S., et al. Sacituzumab Govitecan in Hormone Receptor-Positive/Human Epidermal Growth Factor Receptor 2-Negative Metastatic Breast Cancer. *J. Clin. Oncol.*, **40**: 3365-3376 (2022).
17. Tagawa, S.T., et al. TROPHY-U-01: A Phase II Open-Label Study of Sacituzumab Govitecan in Patients With Metastatic Urothelial Carcinoma Progressing After Platinum-Based Chemotherapy and Checkpoint Inhibitors. *J. Clin. Oncol.,* **39**: 2474-2485 (2021).
18. Etienne-Grimaldi, M.C., et al., UGT1A1 genotype and irinotecan therapy: general review and implementation in routine practice. *Fundam. Clin. Pharmacol*., **29**: 219-237 (2015).
